# Photocatalytic formation of carbon–sulfur bonds

**DOI:** 10.3762/bjoc.14.4

**Published:** 2018-01-05

**Authors:** Alexander Wimmer, Burkhard König

**Affiliations:** 1 Department of Chemistry and Pharmacy, Institute of Organic Chemistry, University of Regensburg, Universitätsstraße 31, 93053 Regensburg, Germany

**Keywords:** disulfides, photocatalysis, sulfones, sulfoxides, thiols, visible light

## Abstract

This review summarizes recent developments in photocatalyzed carbon–sulfur bond formation. General concepts, synthetic strategies and the substrate scope of reactions yielding thiols, disulfides, sulfoxides, sulfones and other organosulfur compounds are discussed together with the proposed mechanistic pathways.

## Introduction

Visible-light photoredox catalysis has developed into an important tool for organic synthesis in the last two decades. Energy-efficient and cheap visible-light-emitting diodes are perfect light sources allowing chemists now to conduct photocatalyzed reactions without special or expensive equipment. Photoredox-active metal complexes or organic dyes are used to initiate photo-induced single-electron transfer (SET) processes upon excitation with visible-light. Such photooxidations or photoreductions yield reactive organic radicals, which can undergo unique bond forming reactions, under very mild conditions.

The key concepts of photocatalysis and photoredox-catalyzed reactions for carbon–carbon and carbon–heteroatom (C–X) bond formation have been reviewed in detail. However, several new photocatalytic methods for the formation of carbon–sulfur (C–S) bonds were recently reported and we aim to summarize the current developments of this emerging field of photoredox catalysis [1–13] in this review.

Sulfur-containing molecules play important roles in many areas of chemistry and materials science. Many natural products, drugs, crop-protection chemicals or substances used in material synthesis bear sulfur-containing functional groups. Furthermore, the unique chemical properties of sulfur find applications in asymmetric catalysis or material design [14–19]. Therefore, many well established and highly efficient strategies for the formation of C–S bonds were developed and already excellently reviewed, including nucleophilic substitution reactions between S-nucleophiles and organic electrophiles, metal-catalyzed C–S bond formations and organocatalytic or enzymatic approaches [15–16,18,20–22].

**Scheme 1:**
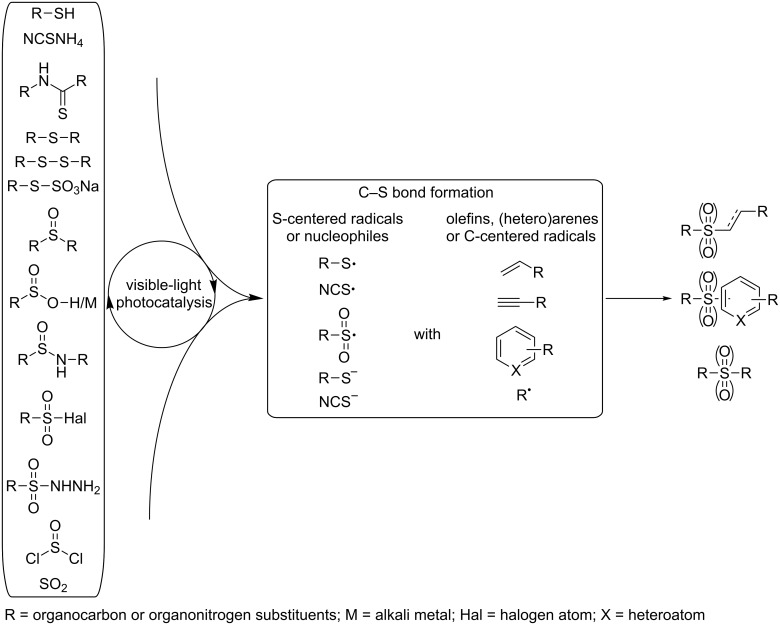
General overview over the sulfur-based substrates and reactive intermediates that are discussed in this review.

This review provides a brief overview over the most important visible-light induced and photoredox-catalyzed approaches for the formation of C–S bonds (Scheme 1). The survey is structured according to the sulfur-containing starting material, beginning with sulfur at its lowest oxidation state – and covers:

- Thiols, thiocyanates, carbon disulfide, thioamide derivatives and sulfides
- Disulfides and thiosulfates
- Sulfoxides
- Sulfinic acids, sulfinate salts and sulfinamides
- Sulfonyl halides, sulfonyl hydrazines, thionyl chloride and sulfur dioxide

C–S bond formations initiated by irradiation with light of wavelengths shorter than 380 nm or by radical initiators as well as photocatalytic reactions, where sulfur-containing substrates act as a sacrificial agent are not discussed in this review [23–28].

## Review

### Thiols

#### Formation of sulfides and sulfoxides

**Scheme 2:**
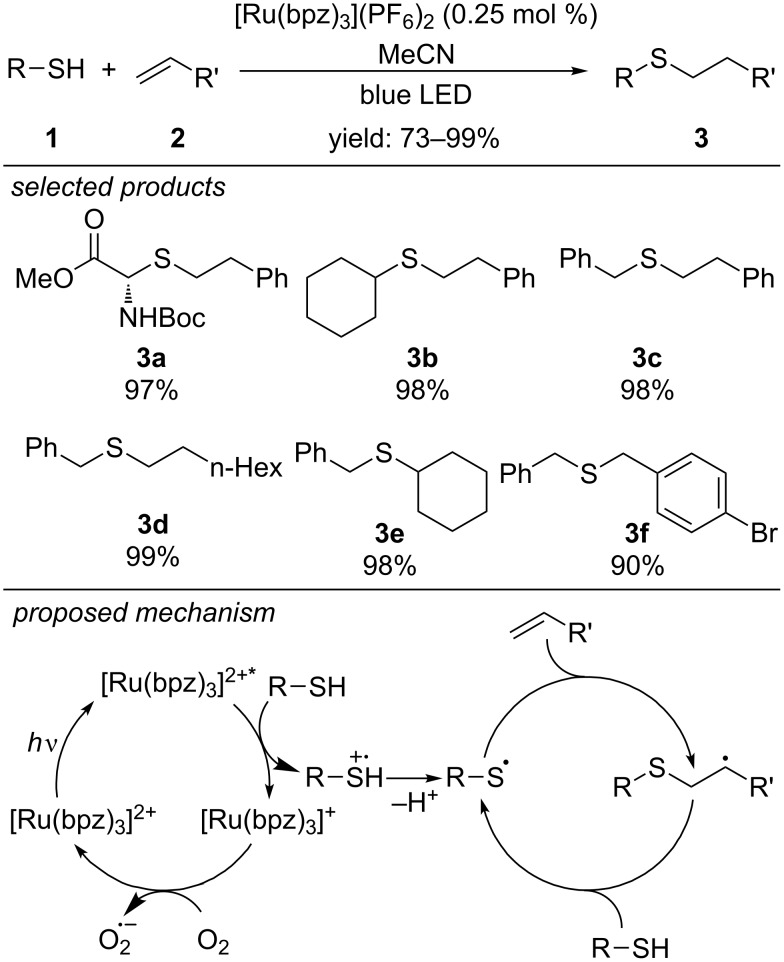
Photoredox-catalyzed radical thiol–ene reaction, applying [Ru(bpz)_3_](PF_6_)_2_ as photocatalyst.

A large number of photocatalytic C–S bond-forming methods report the preparation of sulfides. Non-photocatalytic procedures apply the so-called radical thiol–ene or radical thiol–yne reactions for efficient cross-coupling of thiols with olefins [24,29–30]. In 2013, Yoon and co-workers developed a photoredox-catalyzed version of the radical thiol–ene reaction (Scheme 2) [31]. The thiyl radical was generated as reactive key intermediate from a variety of thiols by photooxidation using [Ru(bpz)_3_](PF_6_)_2_. Aliphatic and aromatic thiols react with aliphatic and aromatic alkenes and alkynes in high to excellent yields to the anti-Markovnikov addition adducts. However, an excess of 4 equivalents of the thiol is needed for the reaction. The authors addressed this limitation in a later report (vide infra). Functional groups like alcohols, Boc-protected amines, carbonyls, esters and halides are tolerated.

**Scheme 3:**
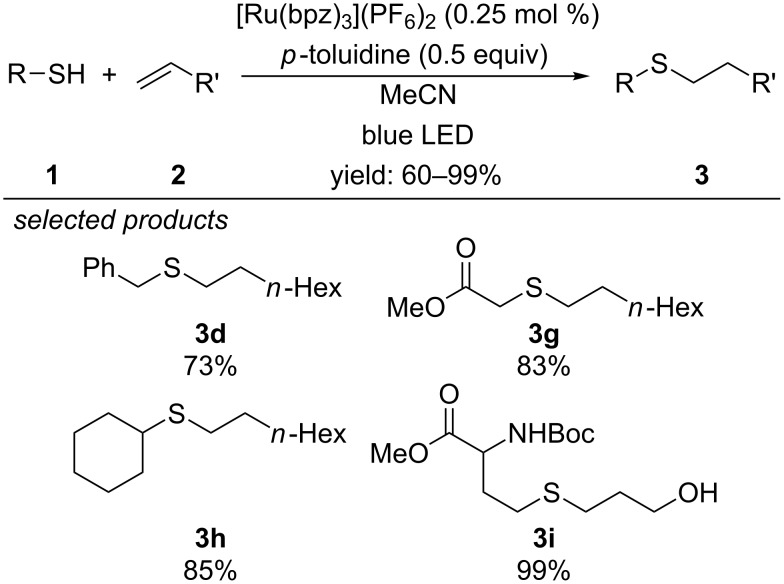
Photoredox-catalyzed thiol–ene reaction of aliphatic thiols with alkenes enabled by aniline derivatives as redox mediators.

One year later, Yoon and co-workers described a redox mediator concept, which was applied to their photocatalytic radical thiol-ene reaction (Scheme 3) [32]. While the photooxidation of aliphatic thiols by excited [Ru(bpz)_3_](PF_6_)_2_ is thermodynamically feasible, this process is kinetically hindered. Therefore, relatively low yields are observed or the amount of thiol has to be increased. To overcome this limitation, anilines were used as redox mediators, which are first oxidized by the photocatalyst and subsequently activate the aliphatic thiol via direct hydrogen abstraction or sequential electron- and proton-transfer steps. With this concept they were now able to apply cysteine-containing glutathione for the reaction with a series of highly functionalized alkenes to form the respective sulfide adducts with yields up to 99%.

**Scheme 4:**
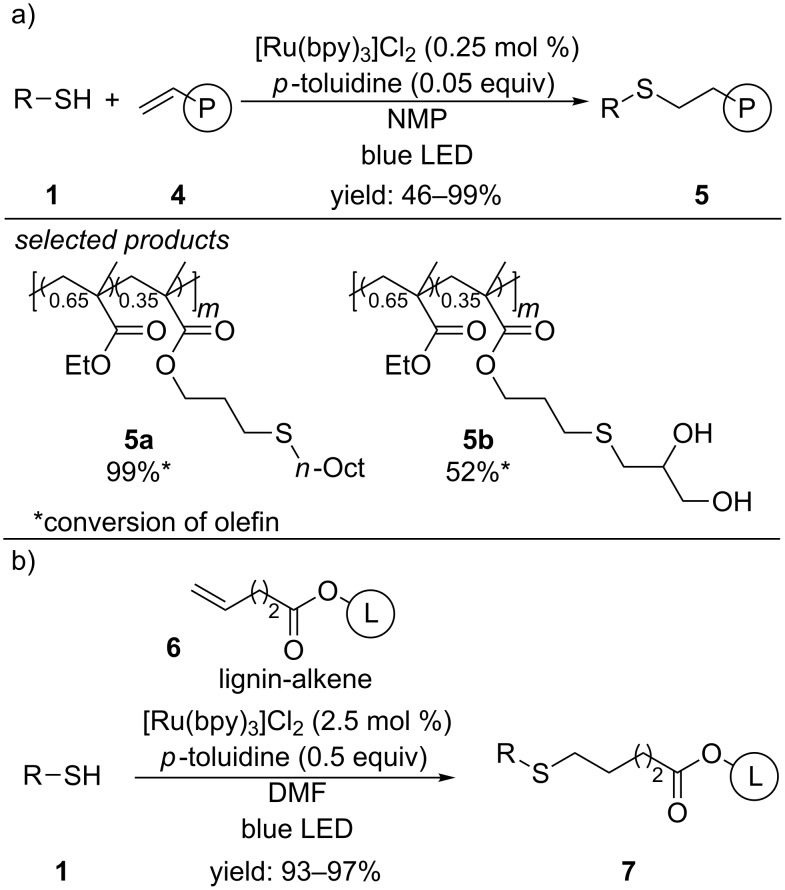
Photoredox-catalyzed radical thiol–ene reaction for the postfunctionalization of polymers (a) and natural lignin (b).

This concept attracted attention from different fields of chemistry. Boyer and co-workers for example applied the redox mediator accelerated photoredox-catalyzed radical thiol–ene reaction for polymer postfunctionalization and step-growth addition polymerization (Scheme 4a) [33]. In contrast to Yoon’s conditions, they used [Ru(bpy)_3_]Cl_2_ as photocatalyst and *N*-methyl-2-pyrrolidone as solvent and were able to efficiently couple polybutadiene and poly(allyl methacrylates) with a series of functionalized thiols. Step-growth addition polymerization for the preparation of linear polymers also was achieved using dithiols and dienes for the reaction.

Recently, Chung and co-workers were successful in functionalizing natural lignin by applying Yoon’s concept [32] (Scheme 4b) [34]. They first introduced alkene moieties to the chemically inert lignin structure by esterification of the hydroxy groups of lignin with 4-pentenoic acid. Subsequent radical thiol–ene reaction with aliphatic thiols, using [Ru(bpy]_3_Cl_2_ as photocatalyst and *p*-toluidine as redox mediator afforded high yields of the desired lignin derivatives.

**Scheme 5:**
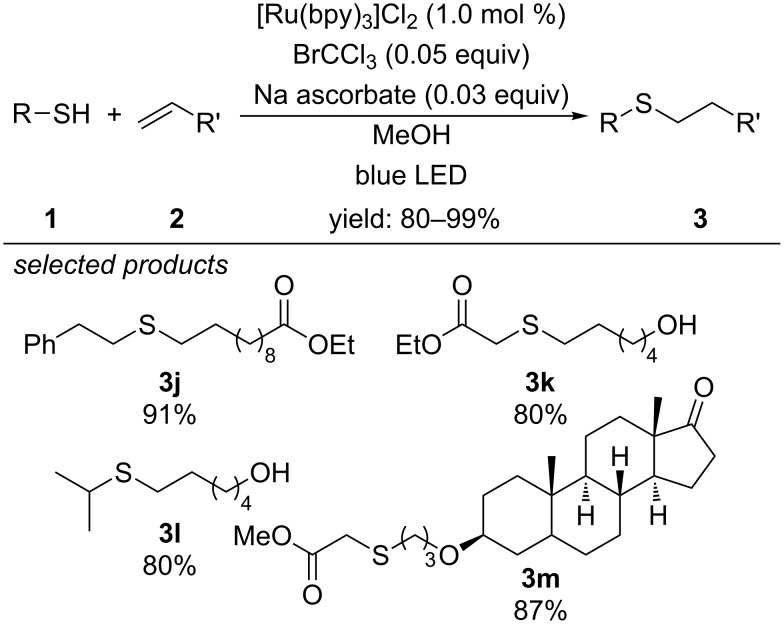
Photoredox-catalyzed thiol–ene reaction enabled by bromotrichloromethane as redox additive.

In 2014, Stephenson and co-workers developed a similar concept for the photocatalytic radical thiol–ene reaction (Scheme 5) [35]. Instead of using aniline derivatives as redox mediators for the activation of thiols, they generated the trichloromethyl radical by single-electron reduction of bromotrichloromethane. Subsequent hydrogen atom abstraction from the thiol yields chloroform and the reactive thiyl radical, which then undergoes radical thiol–ene coupling. The scope comprises the coupling of alkyl, acyl and benzyl thiols with alkenes. The coupling of thiols with alkynes gave 1,2-dithioether.

**Scheme 6:**
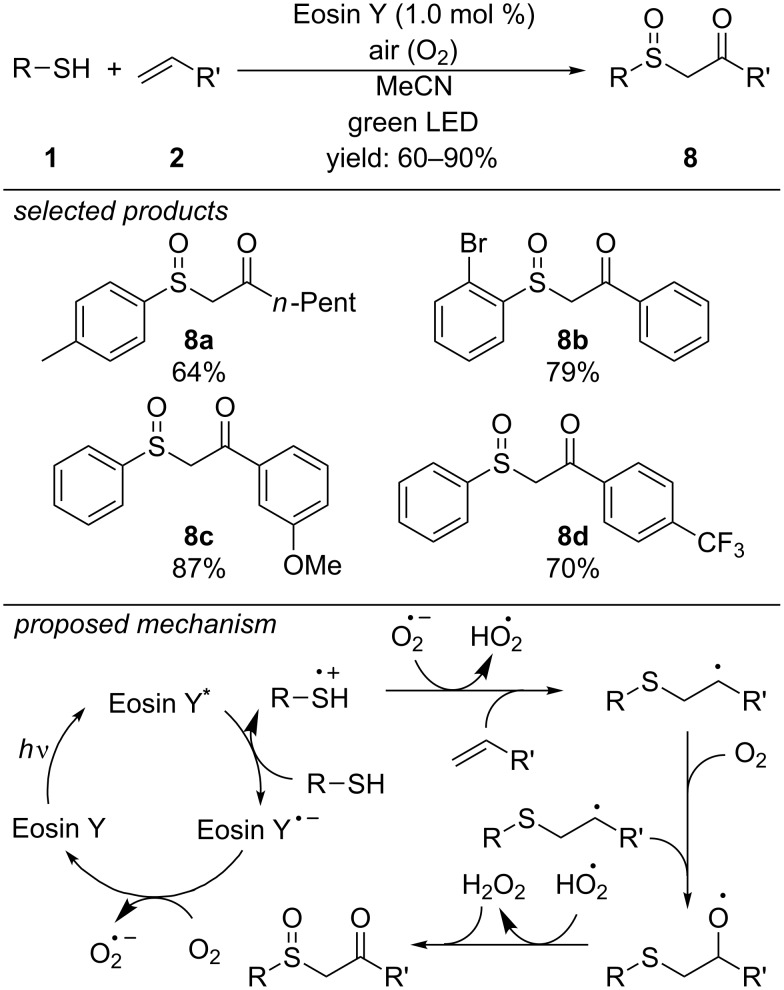
Photoredox-catalyzed preparation of β-ketosulfoxides with Eosin Y as organic dye as photoredox catalyst.

Later that year, Yadav and co-workers reported a new photoredox-catalyzed method for the preparation of β-ketosulfoxides (Scheme 6) [36]. The reaction proceeds again via a radical thiol–ene pathway, but oxygen plays a key role in this reaction. The authors propose that the photo-excited state of the organic dye Eosin Y is reductively quenched by the aryl thiol to form the Eosin Y radical anion and the respective aryl thiyl radical cation. Neutral Eosin Y is regenerated through oxidation of the radical anion by dioxygen. The resulting superoxide radical anion then deprotonates the thiyl radical cation. Subsequent addition to the alkene yields the anti-Markovnikov radical intermediate. Radical addition to dioxygen leads finally to the β-ketosulfide, which subsequently is oxidized by the in situ generated hydrogen peroxide radical to the respective β-ketosulfoxide. No additional sacrificial substrates are needed in order to regenerate the photocatalyst or for the oxidation of the sulfenyl intermediate to the respective sulfoxide moiety. Hydrogen peroxide, which is generated as a byproduct, directly is consumed by oxidizing the sulfide to the sulfoxide. Different aryl thiols were reacted with aliphatic and aromatic alkenes, showing a reasonable functional group tolerance.

**Scheme 7:**
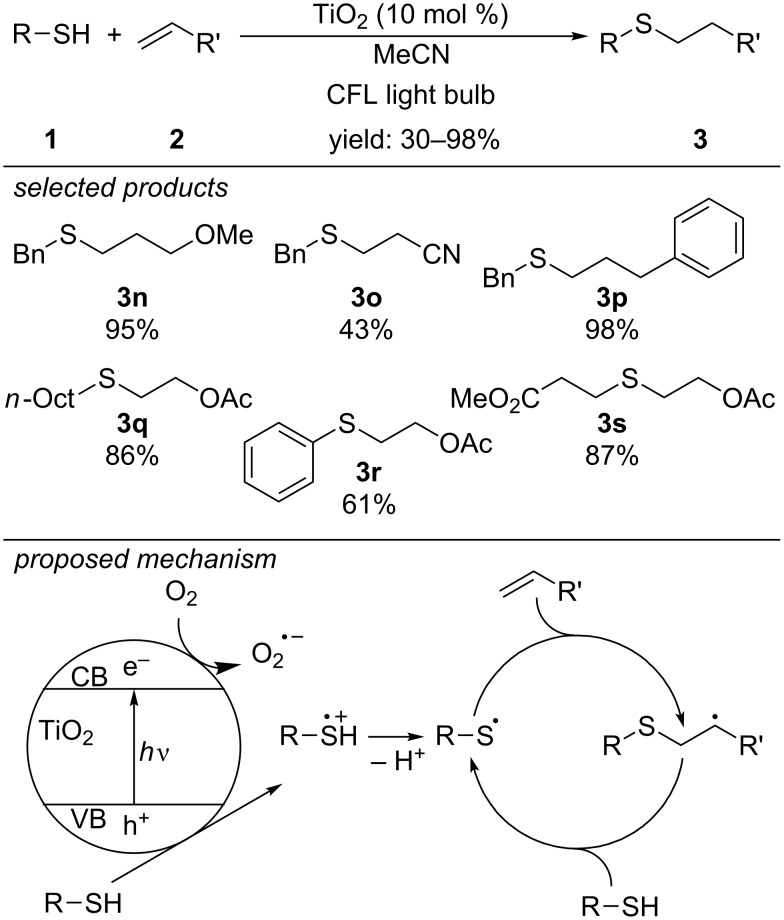
Greaney’s photocatalytic radical thiol–ene reaction, applying TiO_2_ nanoparticles as photocatalyst.

In 2015, the group of Greaney substituted the transition metal photocatalysts by titanium dioxide (TiO_2_) nanoparticles (Scheme 7) [37]. Photoexcitation of electrons to the conduction band of TiO_2_ leads to electron holes in the valence band, which can be reductively quenched by the thiol to form the respective thiyl radical cation. After deprotonation, the thiyl radical undergoes thiol–ene coupling. The scope of the reaction includes the coupling of primary alkyl and aryl thiols with primary and 1,1-disubstituted alkenes.

**Scheme 8:**
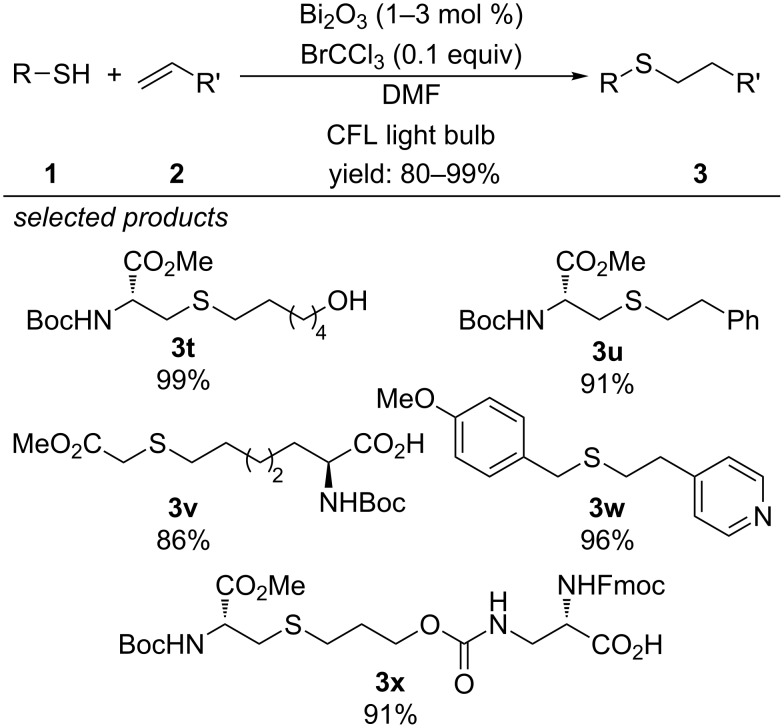
Fadeyi’s photocatalytic radical thiol–ene reaction, applying Bi_2_O_3_ as photocatalyst.

In the same year, Fadeyi et al. reported bismuth oxide (Bi_2_O_3_) as photoredox catalyst in combination with bromotrichloromethane as redox additive (Scheme 8) [38]. A series of alkyl and benzyl thiols were reacted with aliphatic alkenes and styrenes. The method tolerates the presence of pyridine derivatives, alcohols, esters, carboxylic acids, Boc-protected amines, boronic pinacol esters and was applied to the late-stage functionalization of complex molecules.

**Scheme 9:**
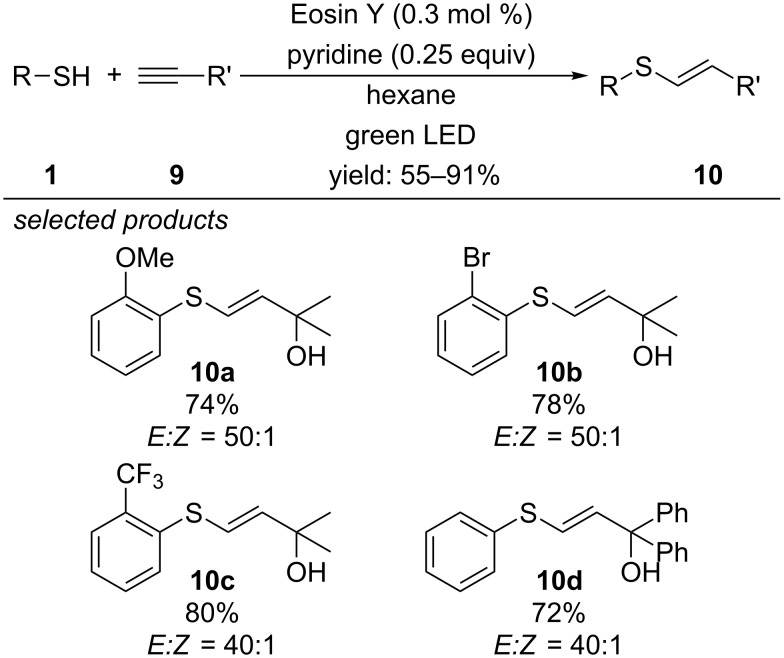
Ananikov’s photocatalytic radical thiol-yne reaction, applying Eosin Y as photocatalyst.

In 2016, the first metal-free photoredox-catalyzed radical thiol–yne reaction was reported by Ananikov and co-workers (Scheme 9) [39]. Their aim was to develop a photocatalytic method that is applicable for the industrial synthesis of pharmaceuticals or bioactive compounds. Using metal-based photocatalysts may leave traces of metal impurities in the crude product causing elaborative purification steps. Applying the organic dye Eosin Y as photocatalyst avoids this problem. The single-electron oxidation of aryl thiols by the excited state of Eosin Y is thermodynamically feasible and forms a thiyl radical cation, which subsequently can be deprotonated by pyridine to the respective thiyl radical. Radical addition to the alkyne leads to the anti-Markovnikov adduct via the more stable secondary radical intermediate. As alkyne 2-methyl-3-butyn-2-ol was selected, which is easily prepared from acetylene and acetone on large scale and the respective thiol–yne adducts can be converted into valuable sulfenylated dienes by dehydration. A series of aryl thiols with different steric and electronic properties give high yields of the thiol–yne products. Noteworthy is the high *E*-selectivity of the resulting alkene. Dependent on the substitution pattern of the aryl thiol a ratio up to 60:1 was observed due to steric effects.

**Scheme 10:**
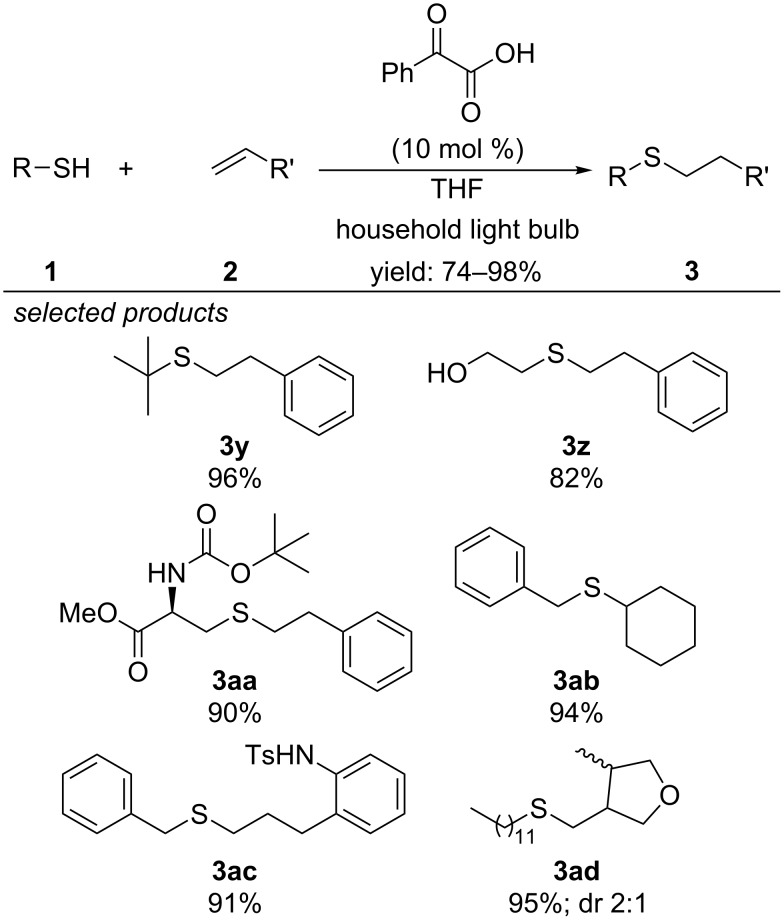
Organocatalytic visible-light photoinitiated thiol–ene coupling, applying phenylglyoxylic acid as organophotocatalyst.

In 2017, the group of Kokotos described a organocatalytic photoinitiated thiol–ene coupling reaction, applying phenylglyoxylic acid as photoorganocatalyst (Scheme 10) [40]. They have shown that the reaction mainly proceeds via a radical chain propagation mechanism, which is initiated by visible-light irradiation of the photoorganocatalyst. Aliphatic and aromatic thiols reacted with aliphatic olefins and styrene derivatives in high yields.

**Scheme 11:**
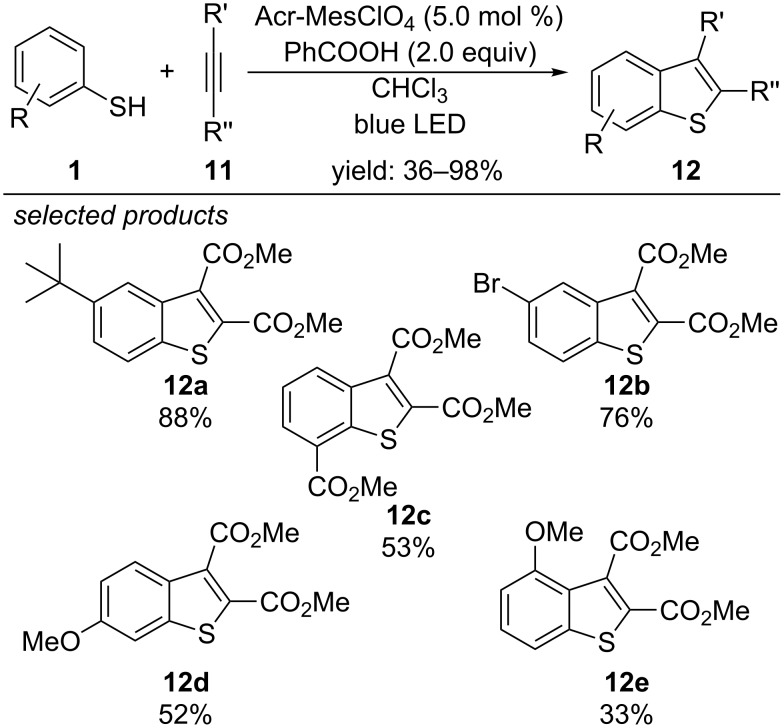
Xia’s photoredox-catalyzed synthesis of 2,3-disubstituted benzothiophenes, applying 9-mesityl-10-methylacridinium perchlorate as organic photocatalyst.

Recently, additional procedures for the photoredox-catalyzed radical thiol–ene and thiol–yne reaction were reported. Xia and co-workers describe a metal-free method for the synthesis of benzothiophenes via a photocatalyzed tandem addition/cyclization reaction (Scheme 11) [41]. Aryl thiols were coupled with dimethyl acetylenedicarboxylate, applying 9-mesityl-10-methylacridinium perchlorate (Acr^+^-Mes ClO_4_^−^) as organic photocatalyst and benzoic acid as oxidant. Based on radical trapping experiments, the authors suggest that the reaction might proceed via a radical addition pathway.

**Scheme 12:**
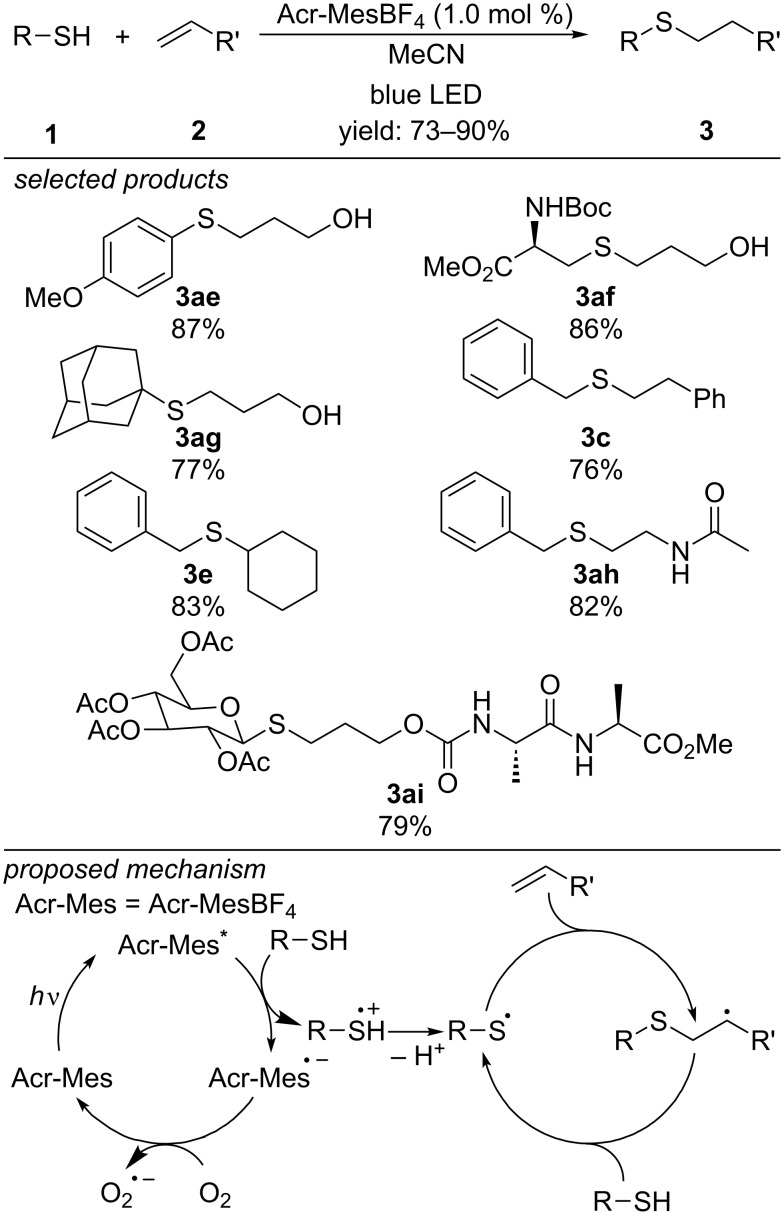
Wang’s metal-free photoredox-catalyzed radical thiol–ene reaction, applying 9-mesityl-10-methylacridinium tetrafluoroborate as organic photocatalyst.

Wang et al. developed a 9-mesityl-10-methylacridinium tetrafluoroborate (Acr^+^-Mes BF_4_^−^) catalyzed radical thiol–ene reaction with broad scope (Scheme 12) [42]. Both linear and branched primary, secondary and aromatic thiols react with linear and substituted aliphatic alkenes and styrenes. Due to the high functional group tolerance, they demonstrated the applicability to the preparation of glycoconjugates from glycosyl thiols and amino acid derivatives. They propose that the photoexcited state of Acr^+^-Mes oxidizes the thiol to the respective thiyl radical cation, which adds to the alkene after deprotonation. The anti-Markovnikov radical intermediate abstracts a hydrogen atom from unreacted thiol, yielding the thiol–ene product and another equivalent of thiyl radical.

**Scheme 13:**
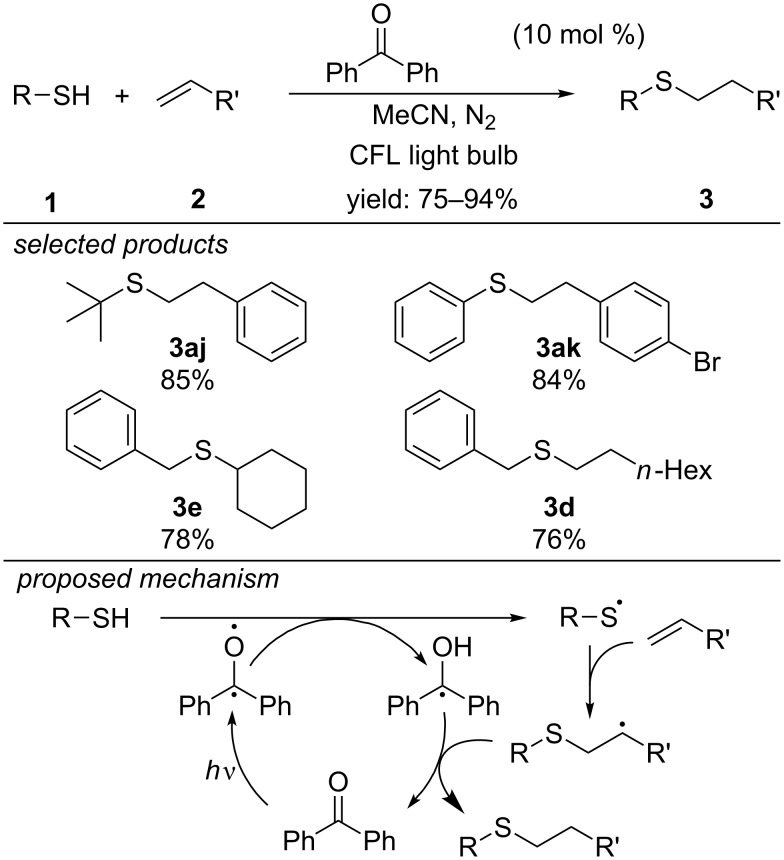
Visible-light benzophenone-catalyzed metal- and oxidant-free radical thiol–ene reaction.

Yadav and co-workers presented a metal-free radical thiol–ene approach, using benzophenone as photoredox catalyst (Scheme 13) [43]. No sacrificial oxidant is required for this reaction as benzophenone is regenerated by hydrogen atom transfer to the anti-Markovnikov radical intermediate. Aliphatic and aromatic thiols react under these conditions with aliphatic alkenes and styrenes.

**Scheme 14:**
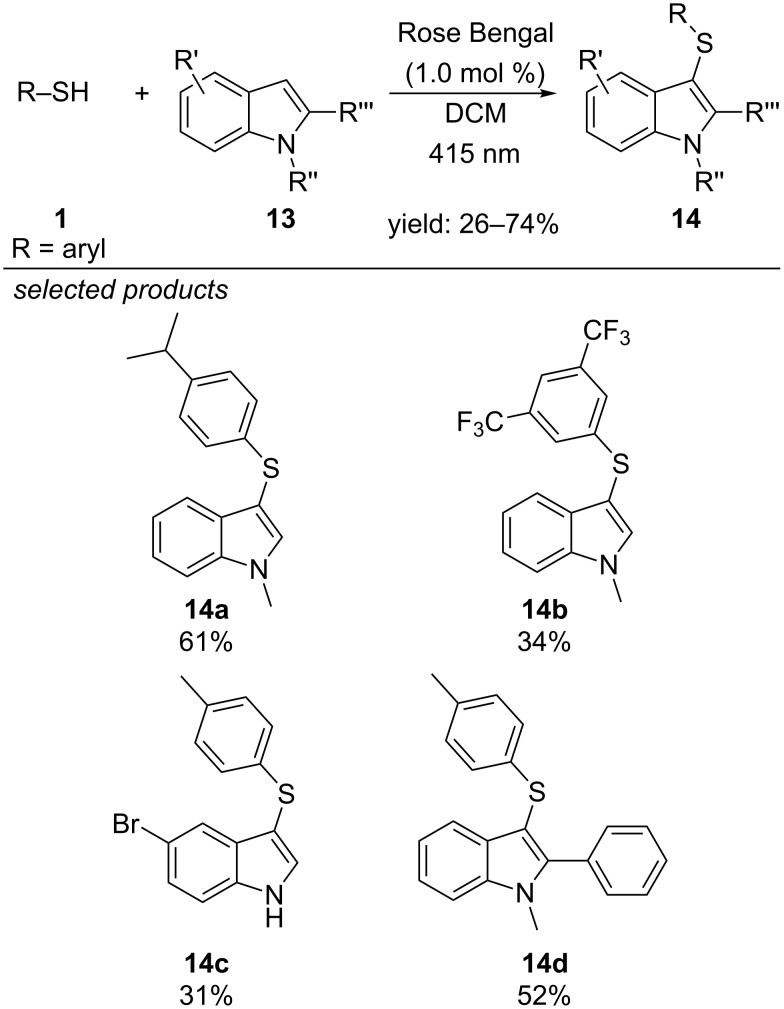
Visible-light catalyzed C-3 sulfenylation of indole derivatives using Rose Bengal as organic dye.

The direct C-3 sulfenylation of indoles with aryl thiols was reported by Guo, Chen and Fan, using Rose Bengal as organic photoredox catalyst and aerobic oxygen as oxidative species (Scheme 14) [44]. They propose that ^1^O_2_ is generated by photoexcited Rose Bengal via energy transfer and abstracts a hydrogen atom from the aryl thiol. Radical addition on the indole derivative, oxidation and rearomatization via deprotonation yields the corresponding sulfenylated indole derivative.

**Scheme 15:**
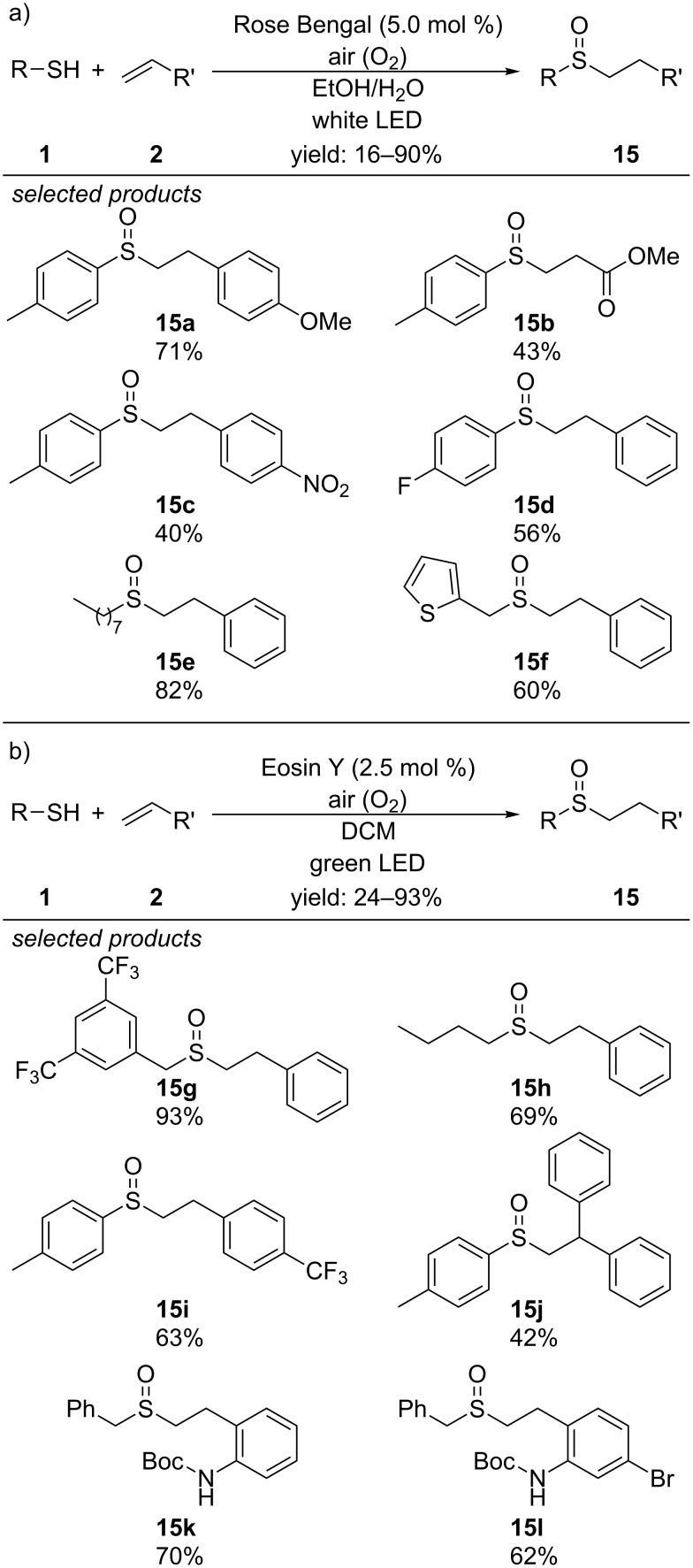
Photocatalyzed radical thiol–ene reaction and subsequent aerobic sulfide-oxidation with Rose Bengal or Eosin Y as organic photocatalysts.

Very recently, Wei, Wang and co-workers and the working groups of Fraile and Aleman independently reported of visible-light photocatalyzed procedures for the preparation of sulfoxides from the respective sulfides and alkenes by radical thiol–ene reactions (Scheme 15) [45–46].

Wei and Wang applied Rose Bengal as organic photocatalyst and were able to couple a series of electron-rich and electron-poor styrene derivatives with aromatic and also long-chain aliphatic thiols to the respective sulfoxides, after aerobic oxidation (Scheme 15a). They describe a photocatalyzed thiol–ene reaction mechanism, where the sulfide-adduct is finally oxidized by an in situ generated superoxide radical anion to form the respective sulfoxide.

In contrast, Fraile, Aleman and co-workers use the organic photocatalyst Eosin Y and propose a different mechanism for their method (Scheme 15b). Based on several quenching experiments, they suggest that after the radical thiol–ene cross-coupling of the thiol with the alkene, the respective sulfide-adduct is oxidized by in situ generated singlet oxygen instead of the superoxide radical anion. Their method was compatible with a similar scope of thiols and alkenes like the one of Wei and Wang.

**Scheme 16:**
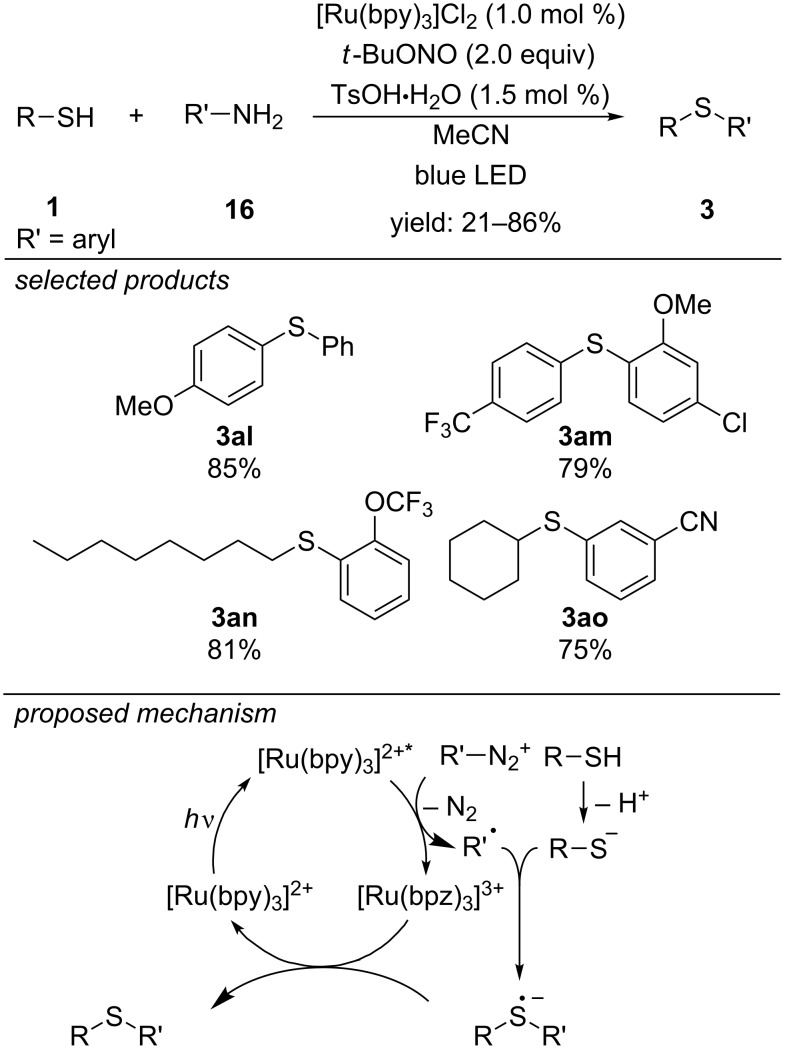
Photoredox-catalyzed synthesis of diaryl sulfides.

A very different strategy for the photoredox-catalyzed preparation of diaryl sulfides was reported in 2013, applying [Ru(bpy)_3_]Cl_2_ as photocatalyst (Scheme 16) [47]. The authors propose a mechanism where in situ generated aryl diazonium salts are cleaved by reduction of the excited state of the photocatalyst to form an aryl radical. This reactive intermediate reacts with the anion of the respective thiol to form the corresponding diaryl sulfide adducts. The reaction conditions tolerate functional groups like alcohols, esters, halides, the trifluoromethyl group and heterocyclic substrates like thiazoles or benzoxazoles.

**Scheme 17:**
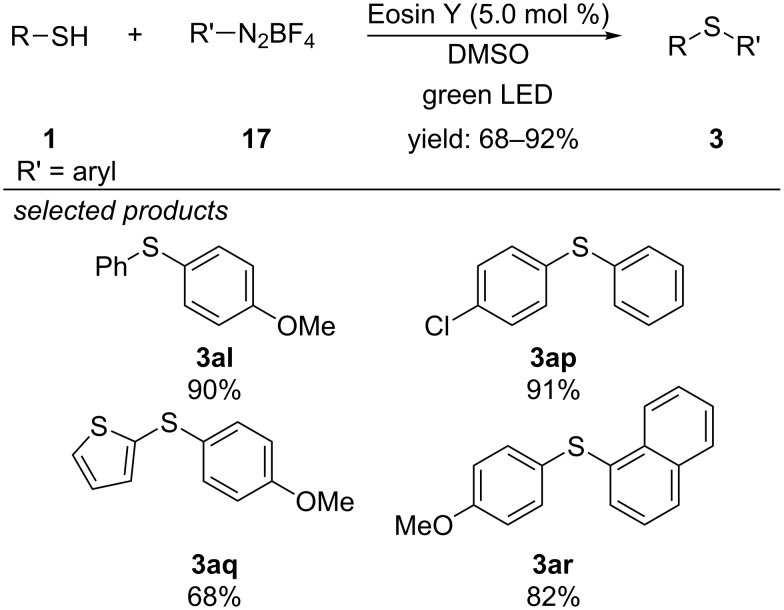
Photocatalytic cross-coupling of aryl thiols with aryl diazonium salts, using Eosin Y as photoredox catalyst.

Starting from aryl thiols and aryl diazonium salts, Lee and co-workers developed a visible-light photocatalyzed procedure for the preparation of diaryl sulfides (Scheme 17) [48]. Applying Eosin Y as organic photocatalyst, both electron-rich and electron-deficient thiols reacted well with various aryl diazonium salts to give the corresponding diaryl sulfide in high yields.

**Scheme 18:**
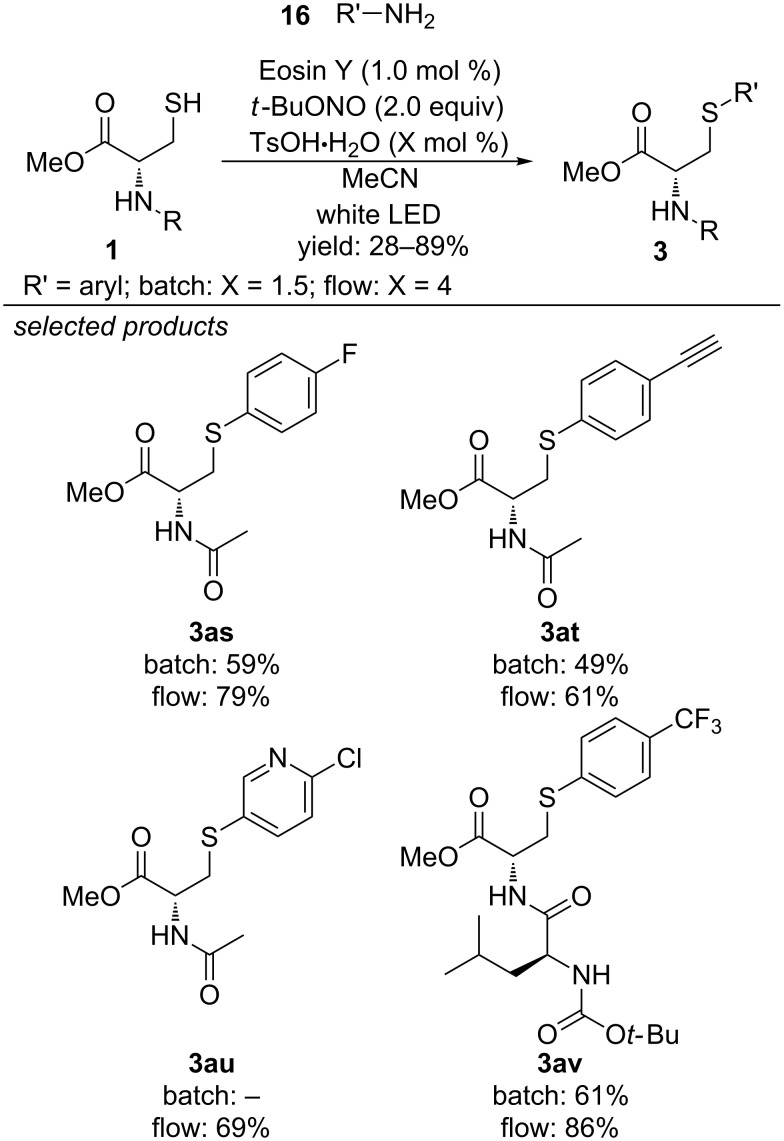
Photocatalyzed cross-coupling of aryl diazonium salts with cysteines in batch and in a microphotoreactor.

Very recently, Noël and co-workers applied the above-mentioned concepts for the selective arylation of cysteine and cysteine-containing peptides in batch as well as in a photomicroreactor (Scheme 18) [49]. They were able to efficiently couple a series of functionalized aryls with the thiol moiety of cysteine, applying the organic photocatalyst Eosin Y. The respective aryl diazonium salts were generated in situ from the respective anilines, *tert*-butyl nitride and catalytic amounts of *p*-toluenesulfonic acid.

**Scheme 19:**
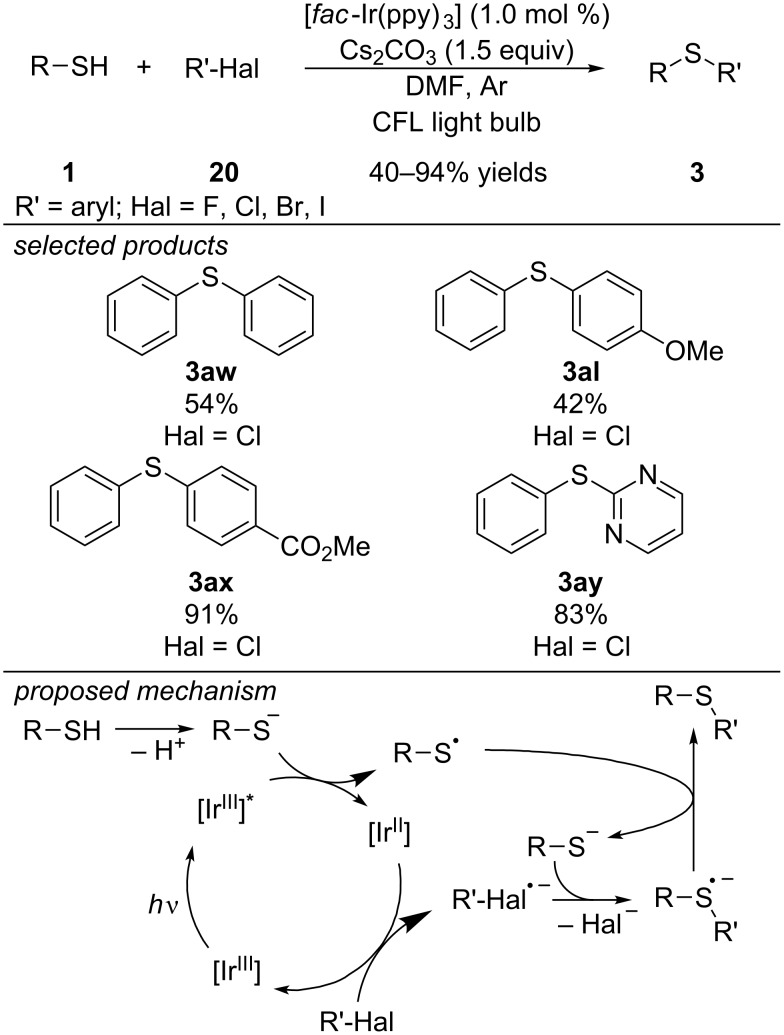
Fu’s [Ir]-catalyzed photoredox arylation of aryl thiols with aryl halides.

Also in 2017, Fu and co-workers reported the C–S cross-coupling of aryl iodides, bromides, fluorides and chlorides with aromatic thiols (Scheme 19) [50]. The reaction is catalyzed by [*fac*-Ir(ppy)_3_]. First, the aromatic thiolate anion, obtained by deprotonation of the thiol with Cs_2_CO_3_ as a base, reductively quenches the photoexcited state of [Ir^III^] to [Ir^II^] and forms a thiyl radical. Next, the [Ir^III^] is regenerated by single-electron reduction of the aryl halide, delivering an aryl halide radical anion. Radical addition to the aromatic thiolate anion forms a sulfide radical anion intermediate, which is oxidized by the photocatalytically generated thiyl radical to give the C–S cross-coupling product.

**Scheme 20:**
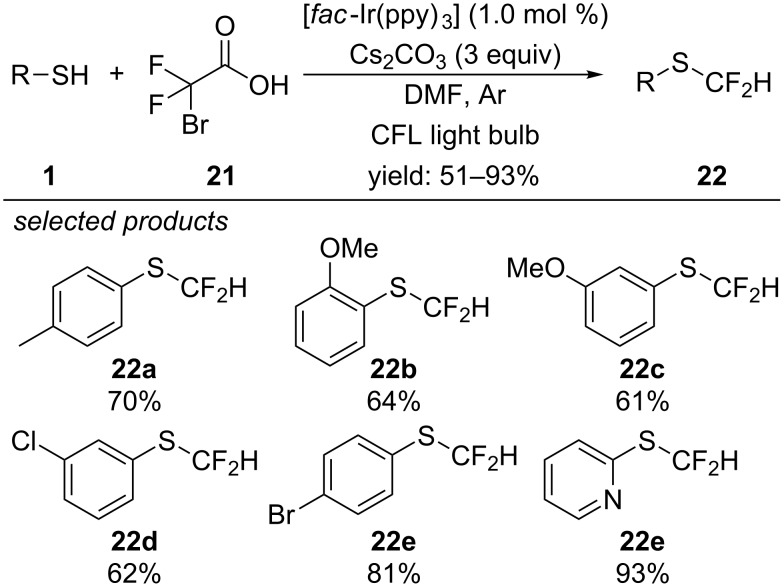
Fu’s photoredox-catalyzed difluoromethylation of aryl thiols.

The same group reported on a photoredox-catalyzed approach for the difluoromethylation of thiophenols, again applying [*fac*-Ir(ppy)_3_] as photocatalyst (Scheme 20) [51]. Herein, they describe a single-electron photoreduction of readily available difluorobromoacetic acid. The respective carbon-centred radical intermediate is then oxidized by the [Ir] catalyst to close the catalytic cycle and form a reactive difluorocarbene intermediate by releasing carbon dioxide. At the same time, the aryl thiol is deprotonated by Cs_2_CO_3_. Finally, the resulting aryl thiol anion reacts with the difluorocarbene to deliver the coupled difluoromethylated aryl sulfide. This method provides a facile synthetic procedure for the selective difluoromethylation of aryl thiols with different functional groups in excellent yields.

**Scheme 21:**
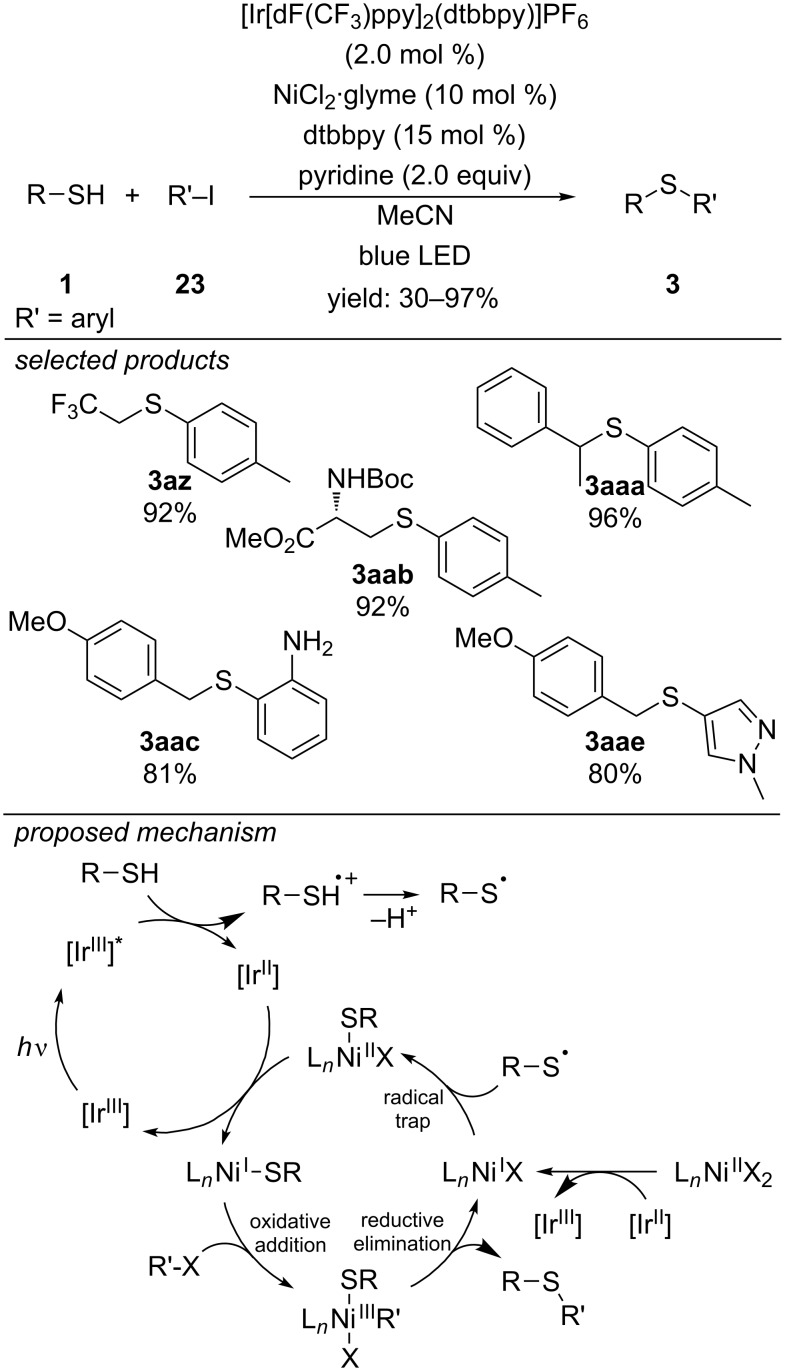
C–S cross-coupling of thiols with aryl iodides via [Ir]-photoredox and [Ni]-dual-catalysis.

A very different approach for the formation of C–S bonds was published by Oderinde, Johannes and co-workers. They combined photoredox catalysis with transition metal catalysis [52–55] for the formation of C–S bonds (Scheme 21) [56]. The reaction proceeds via two different catalytic cycles: The photo-excited state of the [Ir(dF(CF_3_)ppy)_2_(dtbbpy)]PF_6_ photocatalyst is quenched reductively by the thiol forming a thiyl radical cation, which is subsequently deprotonated by pyridine to the respective thiyl radical. The resulting [Ir^II^] species is oxidized to [Ir^III^] by single-electron reduction of the [Ni^II^] catalyst to [Ni^I^]. Fast thiyl radical addition forms a [Ni^II^] intermediate. A second single-electron reduction by [Ir^II^] yields the respective [Ni^I^] sulfide. After oxidative addition of the aryl iodide to the [Ni^I^] sulfide complex, the respective C–S cross-coupling product is formed via reductive elimination from the [Ni^III^] complex, closing the catalytic cycle. Functionalized aryl, benzyl and alkyl thiols cross-coupled with a diverse set of functionalized aryl and heteroaryl iodides, affording the products in up to 97% yield. The reaction proceeds only with aryl iodides, which can be regarded as an advantage for orthogonal organic transformations, but also limits the scope of this method.

**Scheme 22:**
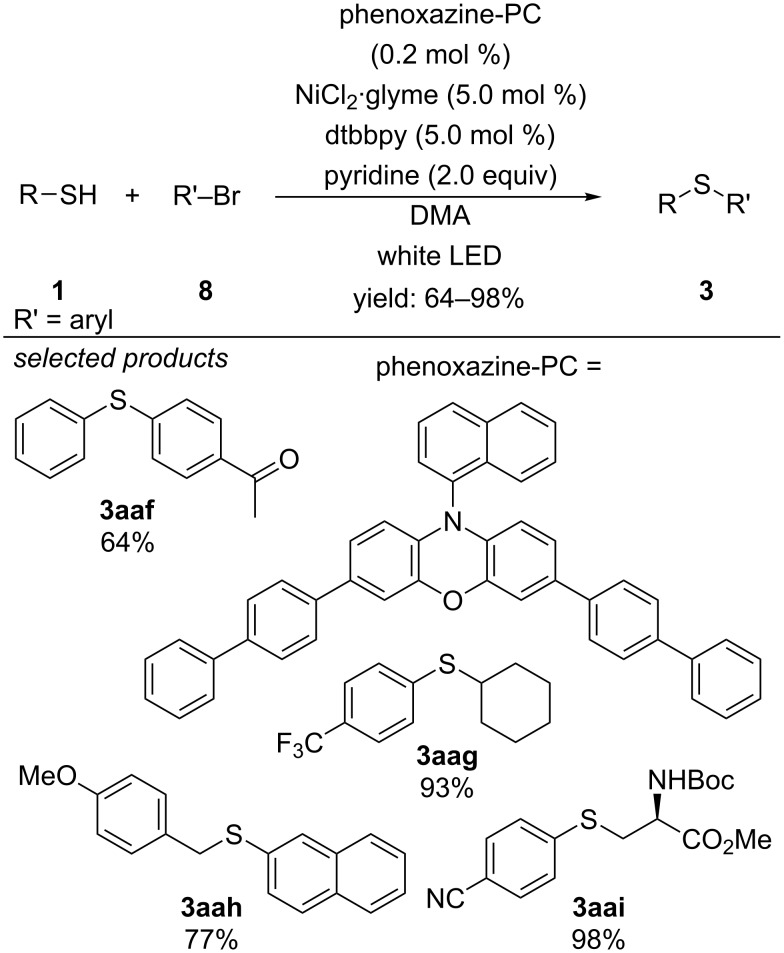
C–S cross-coupling of thiols with aryl bromides, applying 3,7-bis-(biphenyl-4-yl)-10-(1-naphthyl)phenoxazine as organic photocatalyst in combination with transition metal catalysis.

Using 3,7-bis(biphenyl-4-yl)-10-(1-naphthyl)phenoxazine as organic photocatalyst instead of [Ir(dF(CF_3_)ppy)_2_(dtbbpy)]PF_6_, Miyake found out that also aryl bromides could be cross-coupled with a series of different thiols. These were not reactive substrates in the method of Oderinde and Johannes [56] (Scheme 22) [57].

**Scheme 23:**
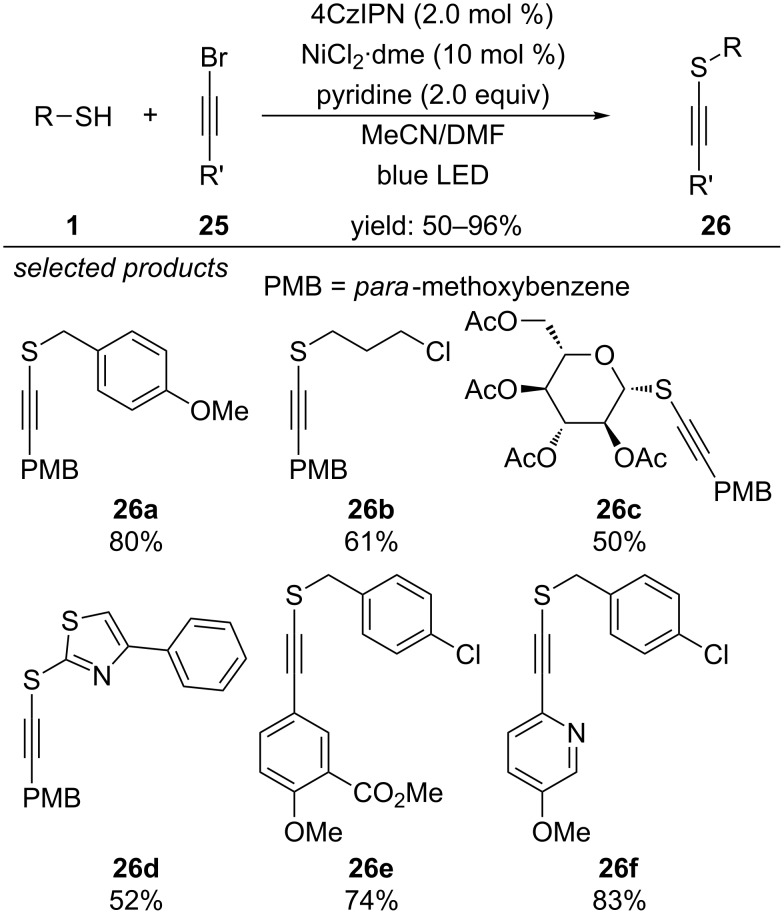
Collin’s photochemical dual-catalytic cross-coupling of thiols with bromoalkynes.

Recently, the laboratory of Collins reported the photochemical dual-catalytic cross-coupling of thiols with bromoalkynes, yielding alkynyl sulfides (Scheme 23) [58]. Applying 4CzIPN (1,2,3,5-tetrakis(carbazol-9-yl)-4,6-dicyanobenzene) as organic photoredox catalyst and [NiCl_2_·dme] as transition metal catalyst in a continuous flow set-up, high yields of the coupling products were obtained in short residence times (30 min). They propose, that photoexcited 4CzIPN* generates the thiyl radical, which adds to the [Ni^I^] complex, yielding a [Ni^II^] species. Single-electron reduction by 4CzIPN^•−^ generates a [Ni^I^] sulfide complex and closes the photocatalytic cycle. Oxidative addition of the bromoalkyne and subsequent reductive elimination forms the corresponding alkynyl sulfide and closes the [Ni]-catalyzed cycle. The reaction conditions were not suitable for aliphatic alkynes as they do not undergo oxidative addition with the [Ni^I^] sulfide complex.

**Scheme 24:**
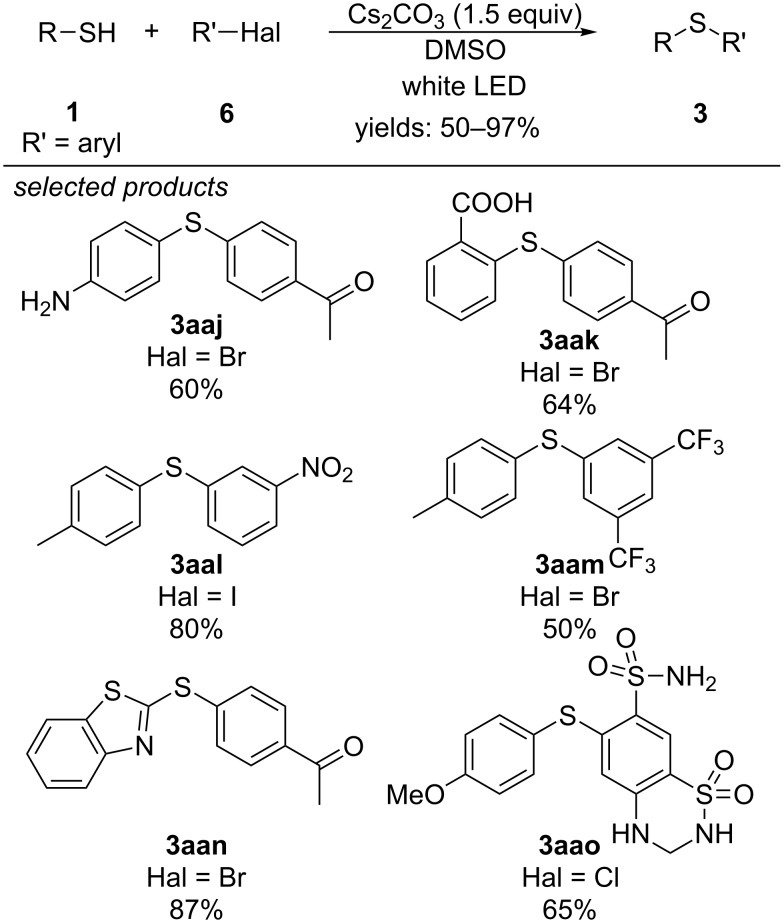
Visible-light-promoted C–S cross-coupling via intermolecular electron donor–acceptor complex formation.

Miyake and co-workers described a very different approach for the C–S cross-coupling between aryl thiols and aryl halides (Scheme 24) [59]. In a mixture with caesium carbonate as a base and DMSO as solvent they observed a colouring of the reaction mixture, whereas the reagents are colourless themselves. They suggest that the thiol becomes deprotonated to the respective thiolate anion and subsequently an electron donor–acceptor complex with the aryl halide is formed. Irradiation with white light then triggers a light-induced electron transfer from the anion to the aryl halide, releasing the halide as an anion and resulting in a thiyl radical and an aryl radical. Finally, radical–radical cross-coupling yields the respective diaryl sulfide.

### Ammonium thiocyanate

#### Formation of thiocyanates

**Scheme 25:**
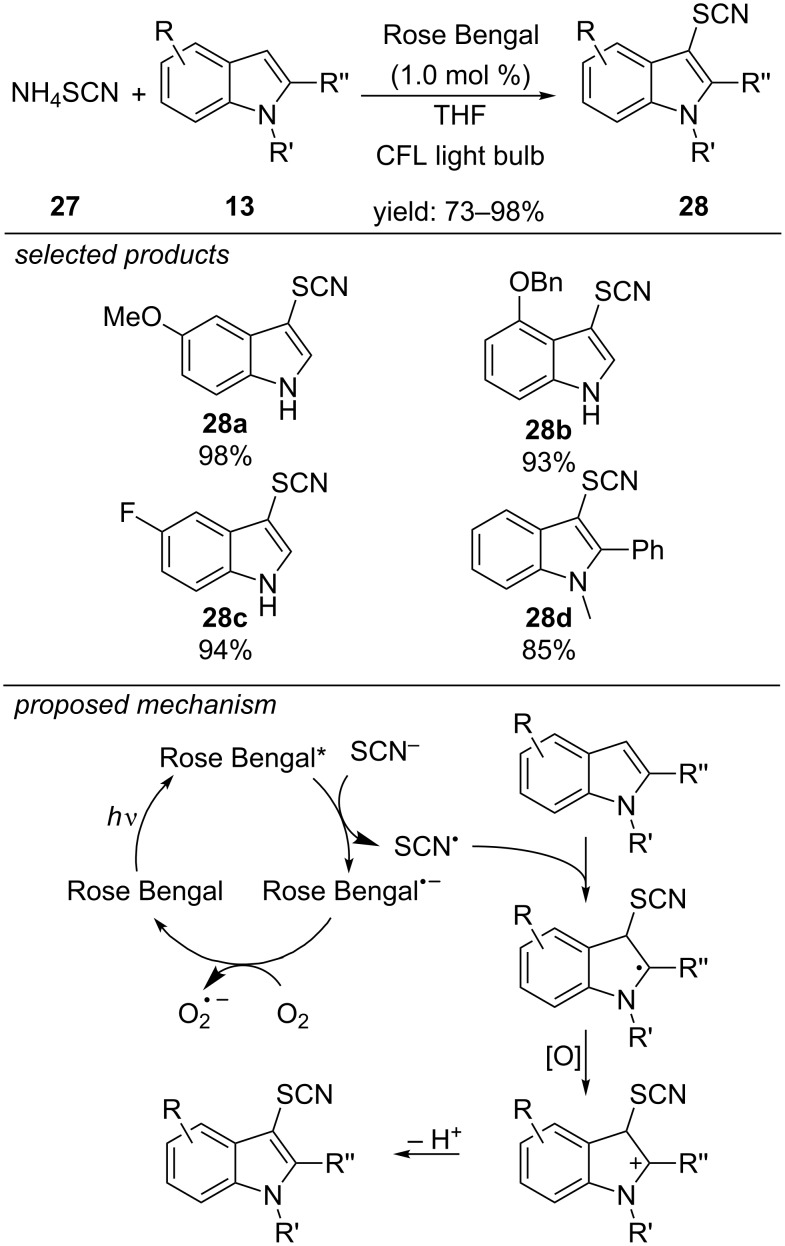
Li’s visible-light photoredox-catalyzed thiocyanation of indole derivatives with Rose Bengal as photocatalyst.

The first photoredox-catalyzed thiocyanation reaction using ammonium thiocyanate as starting material was published in 2014 by Li and co-workers (Scheme 25) [60]. They envisioned that photooxidation of ammonium thiocyanate would lead to a reactive thiocyanate radical. Subsequent radical addition to heteroaromatic substrates like indoles would produce valuable synthetic intermediates. By applying Rose Bengal as organic photocatalyst and aerobic oxygen as terminal oxidant, they were able to functionalize a series of indole derivatives and also could show the applicability of the thiocyanation reaction for gram-scale synthesis with a remarkably low catalyst loading of 0.1 mol %. Oxygen plays an important role in the proposed mechanism: it regenerates the catalyst by aerobic oxidation and oxidizes the carbon-centred radical intermediate, obtained by radical addition of a thiocyanate radical to the indole, to the respective cation. Deprotonation yields the desired thiocyanated indole derivative.

**Scheme 26:**
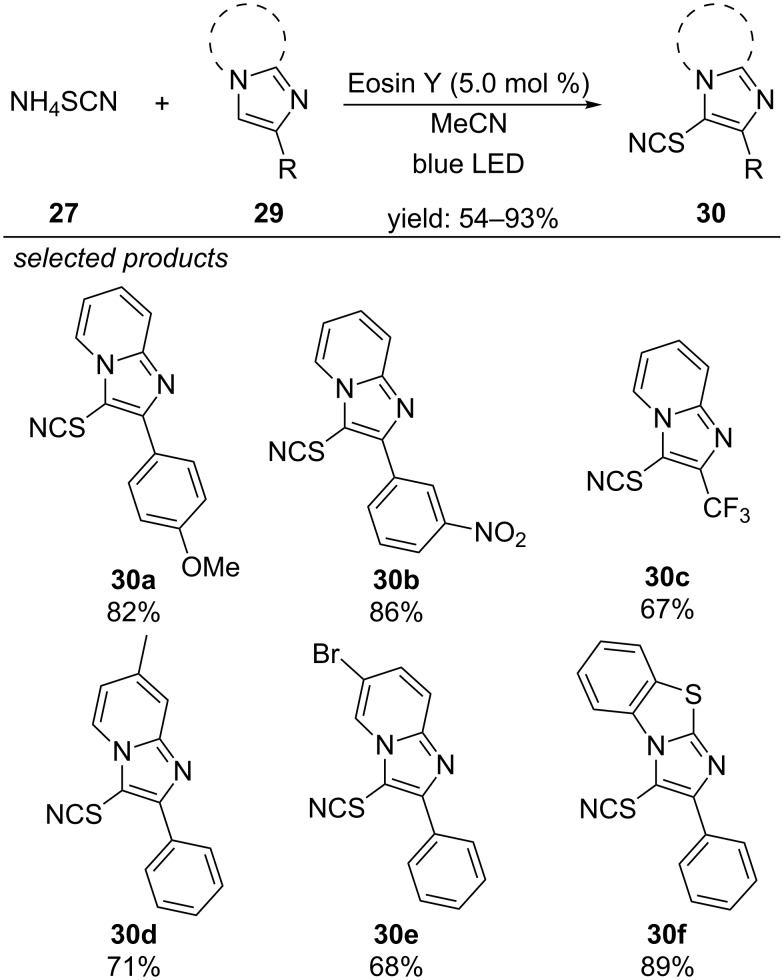
Hajra’s visible-light photoredox-catalyzed thiocyanation of imidazoheterocycles with Eosin Y as photocatalyst.

One year later, the group of Hajra reported a similar approach for the thiocyanation of imidazoheterocycles, applying Eosin Y as photoredox catalyst under aerobic conditions (Scheme 26) [61]. The photoredox-generated thiocyanate radical can add to the heteroaromatic system, which subsequently rearomatizes via deprotonation. This method was suitable for substituted imidazoheterocycles, bearing electron-donating and withdrawing groups. Other imidazole derivatives were unreactive under the reported reaction conditions.

**Scheme 27:**
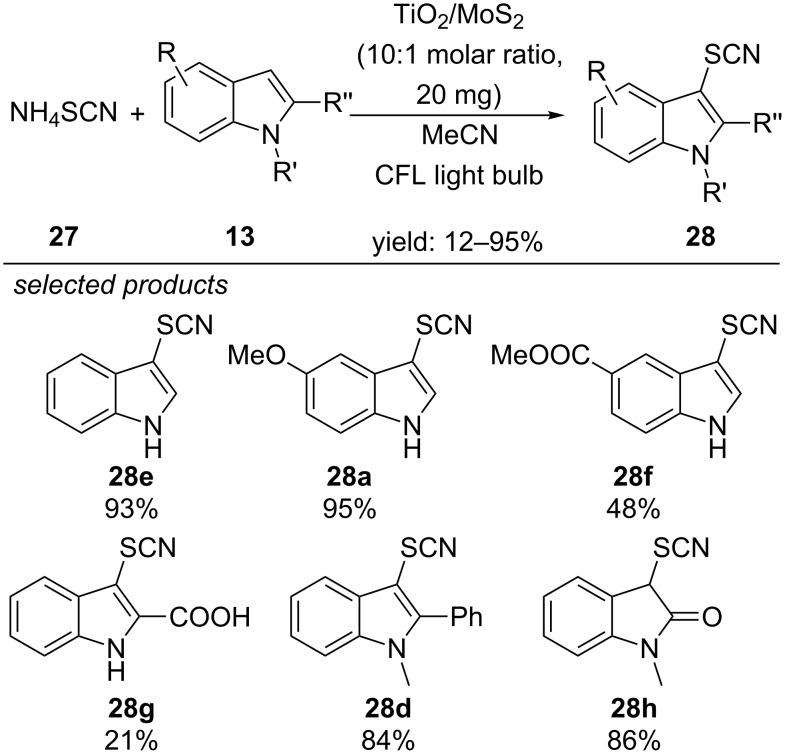
Wang’s photoredox-catalyzed thiocyanation reaction of indoles, applying heterogeneous TiO_2_/MoS_2_ nanocomposite as photocatalyst.

In 2016, Wang and co-workers reported the thiocyanation reaction of indoles with TiO_2_/MoS_2_ nanocomposite as heterogeneous photoredox catalyst and molecular oxygen as terminal oxidant (Scheme 27) [62]. They propose that the efficiency of their reaction is due to a separation of photoinduced electrons and holes. The conduction band of nanoscale MoS_2_ is more positive compared to anatase TiO_2_. Consequently, photoinduced electrons are efficiently transferred from the MoS_2_ conduction band into the lower conduction band of TiO_2_, whereas electron holes are located in the valence band of the nanoscale MoS_2_. The scope of the reaction is quite similar to Li’s report [60]. The photocatalyst nanocomposite was reused in eight consecutive cycles with only slight decrease in activity.

**Scheme 28:**
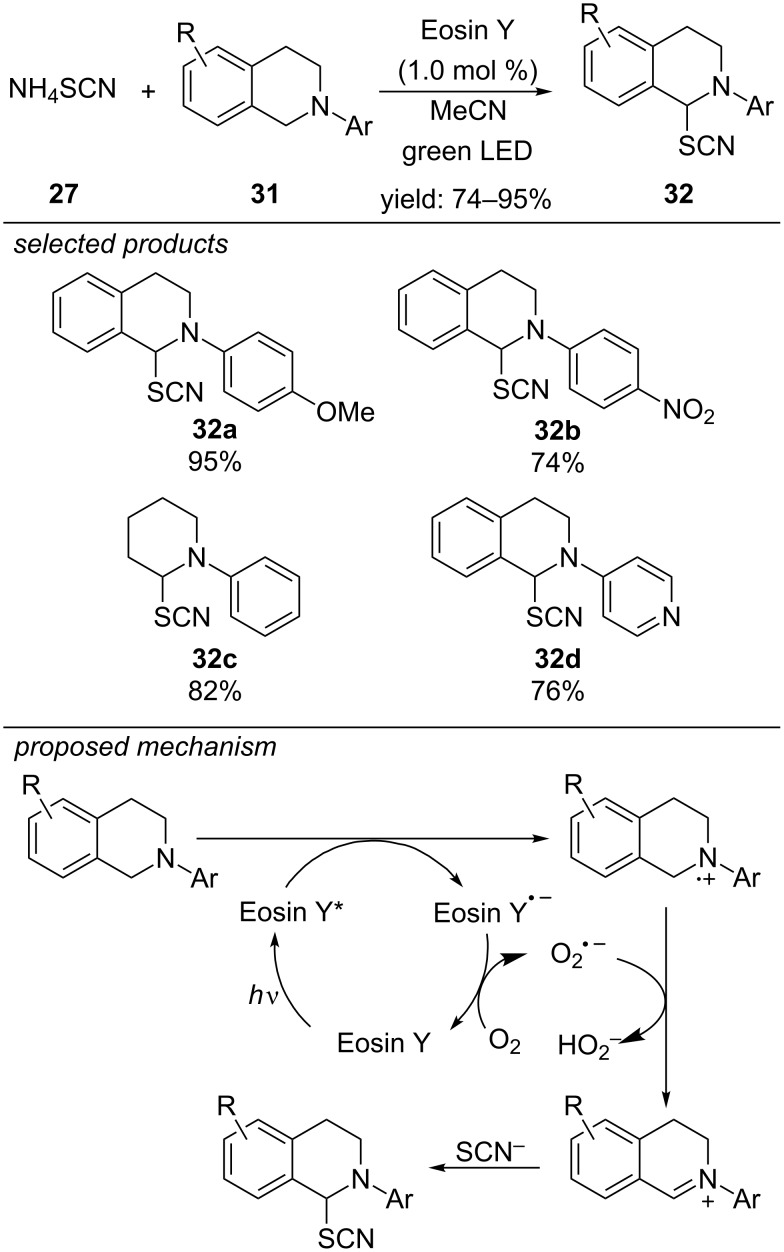
Yadav’s photoredox-catalyzed α-C(sp^3^)–H thiocyanation reaction for tertiary amines, applying Eosin Y as photocatalyst.

Yadav et al. developed a metal-free photoredox-catalyzed α-C(sp^3^)–H thiocyanation reaction for tertiary amines (Scheme 28) [63]. The reaction mechanism is different from the previous examples. The photoexcited state of Eosin Y can be quenched reductively by tertiary amines to form an Eosin Y radical anion and an amine radical cation. Molecular oxygen regenerates Eosin Y and is reduced to its superoxide radical anion. Hydrogen atom abstraction at the α-position of the amine leads to an iminium intermediate, which is attacked by the nucleophilic thiocyanate anion. This strategy was applicable for various substituted 1,2,3,4-tetrahydroisoquinolines and similar derivatives.

#### Formation of 5-aryl-2-imino-1,3-oxathiolanes

**Scheme 29:**
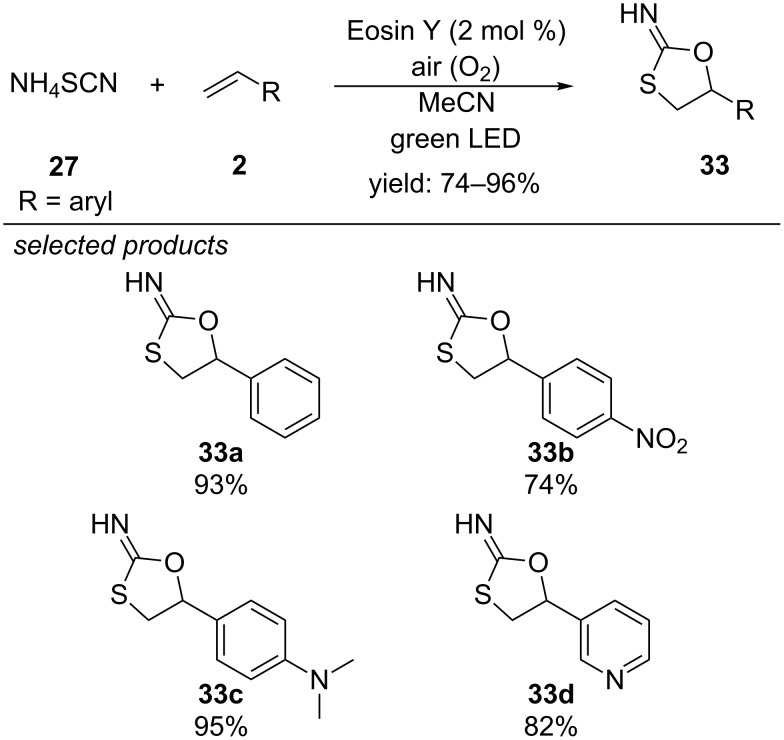
Yadav’s photoredox-catalyzed synthesis of 5-aryl-2-imino-1,3-oxathiolanes.

One year after Li’s report, Yadav and co-workers described a reaction of ammonium thiocyanate (NH_4_SCN) with various styrenes under visible-light catalyzed conditions, utilizing Eosin Y as organic photocatalyst (Scheme 29) [64]. They found that the reaction under air atmosphere resulted in the formation of 5-aryl-2-imino-1,3-oxathiolates, bearing a free imino group. They proposed that the photoexcited state of Eosin Y is reductively quenched by the thiocyanate anion to form the radical anion of Eosin Y and the respective thiocyanate radical. The catalytic cycle is closed by aerobic oxidation of the Eosin Y radical anion. After radical addition of the thiocyanate radical to styrene, the anti-Markovnikov intermediate can form a peroxy radical species with molecular oxygen. Consecutive rearrangements give a β-hydroxythiocyanate, which undergoes fast cyclization to the 5-membered heterocyclic product. The reaction was found to be suitable for a series of steric and electronic different styrenes. However, aliphatic alkenes did not give the desired product. The author’s explain this observation by a lower stability of the free carbon-centred alkyl radical intermediate, compared to benzylic substrates.

### Carbon disulfide

#### Formation of 1,3-oxathiolane-2-thiones

**Scheme 30:**
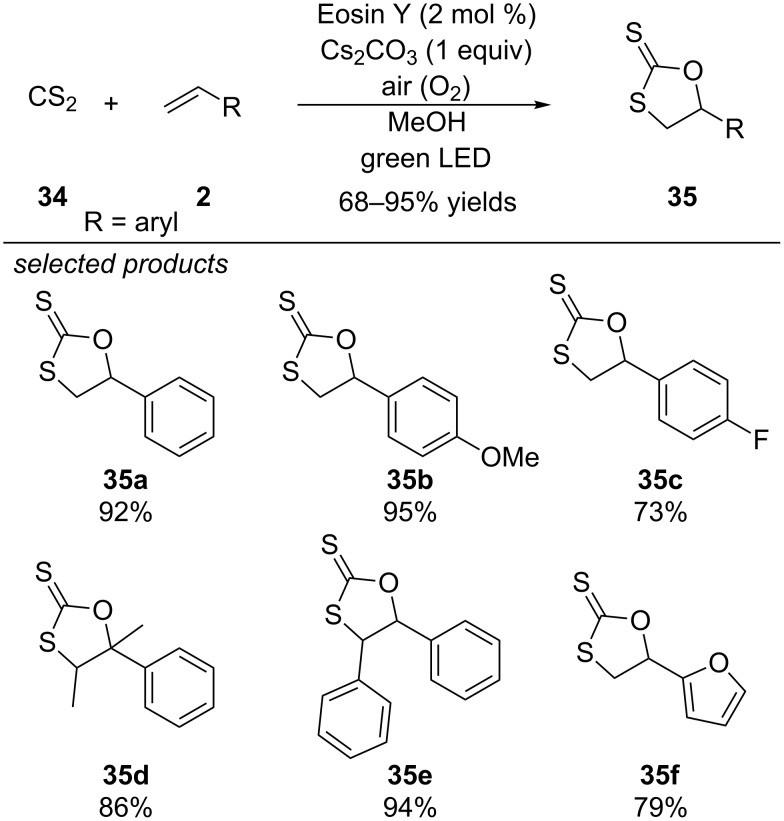
Yadav’s photoredox-catalyzed synthesis of 1,3-oxathiolane-2-thiones.

In 2016, Yadav and co-workers reported a photoredox-catalyzed C–S bond formation with carbon disulfide (CS_2_) as starting material (Scheme 30) [65]. The reaction of CS_2_ with styrenes under visible-light irradiation and Eosin Y as organic photocatalyst was accomplished in the presence of methanol under basic conditions. For a better handling of volatile CS_2_, they converted it into the corresponding caesium methyl xanthate prior to the photocatalytic reaction. Photoexcited Eosin Y is quenched reductively by caesium methyl xanthate, yielding the Eosin Y radical anion. Subsequently, the sulfur-centred radical adds to styrene and forms the 1,3-oxathiolane-2-thione under aerobic conditions. The catalytic cycle is closed by aerobic oxidation of Eosin Y radical anion. Various styrene derivatives are efficiently converted into the corresponding heterocycles. However, aliphatic alkenes could not be applied probably due to the lower radical stability of the carbon-centred alkyl radical intermediate. Nevertheless, the method showed good functional group tolerance to electron-donating and withdrawing groups and also for heterocyclic structures like furans or pyridines.

### Thioamide derivatives

#### Formation of benzothiazoles

**Scheme 31:**
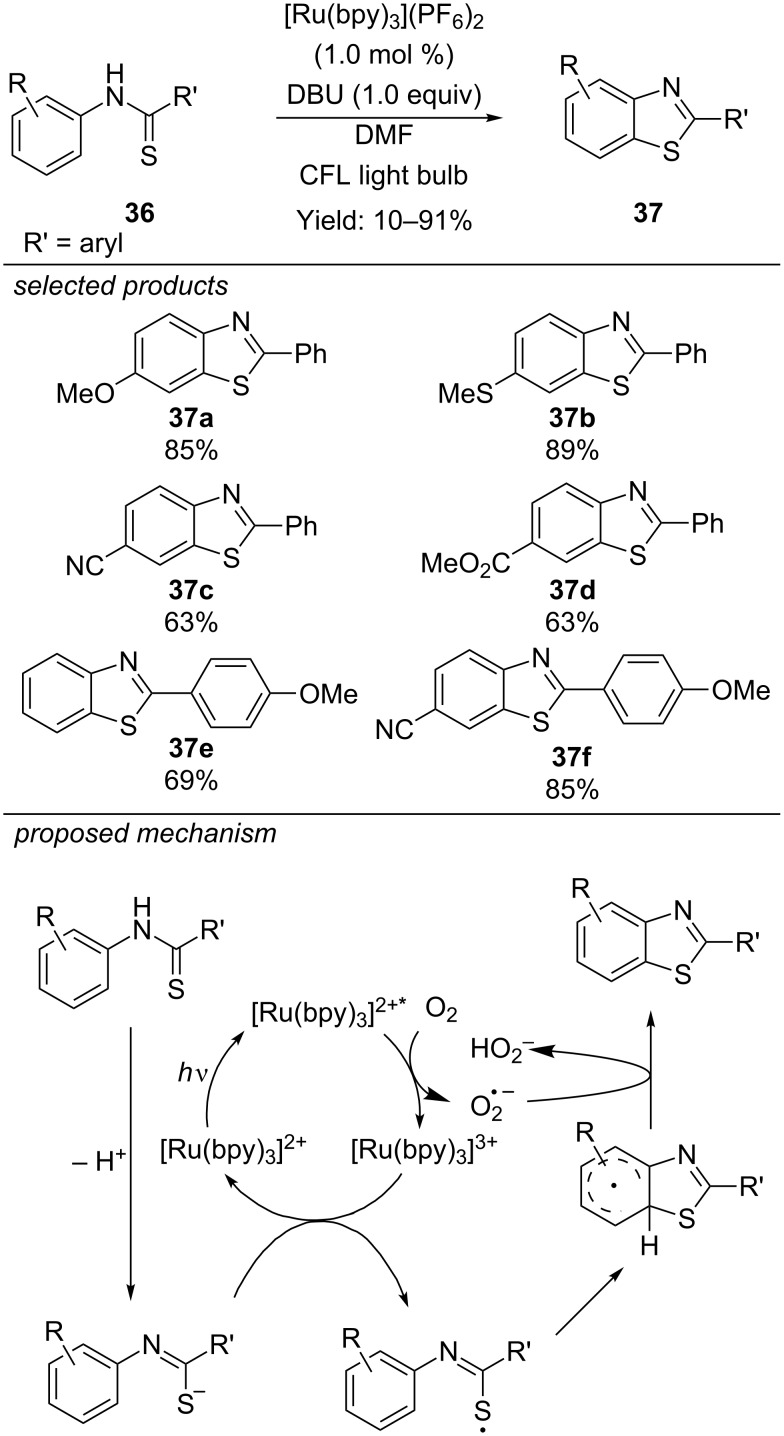
Li’s photoredox catalysis for the preparation of 2-substituted benzothiazoles, applying [Ru(bpy)_3_](PF_6_)_2_ as photocatalyst.

Already in 2012, Li et al. envisioned a photoredox protocol for the synthesis of 2-substituted benzothiazoles applying [Ru(bpy)_3_](PF_6_)_2_ as photocatalyst and molecular oxygen as oxidant (Scheme 31) [66]. As starting material, thiobenzanilides were first deprotonated to the respective sulfur anions. Photoexcited [Ru(bpy)_3_]^2+^* reacts with dioxygen to [Ru(bpy)_3_]^3+^. Subsequent single-electron oxidation of the anion closes the catalytic cycle and generates a thiyl radical, which performs a cycloaddition to the aromatic anilide moiety. Rearomatization via hydrogen atom abstraction by the former generated superoxide radical anion leads to the desired 2-substituted benzothiazole. The authors observed a green colour of the reaction mixture indicating the formation of [Ru(bpy)_3_]^3+^. Therefore, they suggest an oxidative quenching cycle, but an alternative reductive pathway, where photoexcited [Ru(bpy)_3_]^2+^* first oxidizes the sulfur anion by single-electron transfer and is re-oxidized by dioxygen could not be excluded.

**Scheme 32:**
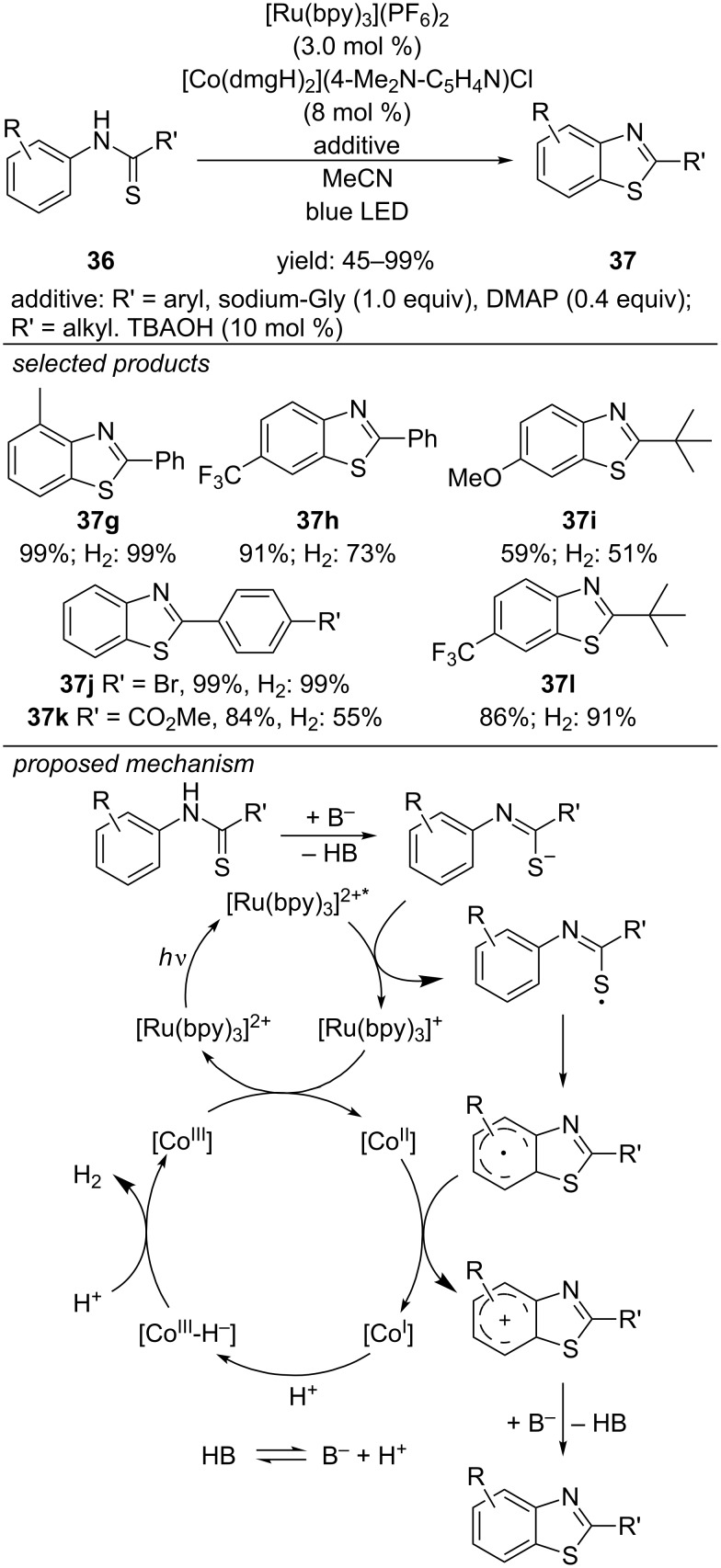
Lei’s external oxidant-free synthesis of 2-substituted benzothiazoles by merging photoredox and transition metal catalysis.

Lei and co-workers reported an external oxidant-free photocatalyzed procedure for the same reaction, also applying [Ru(bpy)_3_](PF_6_)_2_ as photocatalyst (Scheme 32) [67]. Instead of using dioxygen as sacrificial oxidant and basic additives for proton scavenging, they installed a second catalytic cycle for proton reduction deliberating dihydrogen as byproduct. The reaction is proposed to proceed via a reductive quenching of the photoexcited [Ru(bpy)_3_]^2+^* leading to [Ru(bpy)_3_]^+^ and a thiyl radical intermediate. Radical addition to the aromatic anilide moiety, single-electron oxidation and further deprotonation forms the desired 2-substituted benzothiazole by rearomatization. Both the regeneration of the [Ru(bpy)_3_]^2+^ by single-electron oxidation as well as the oxidation and deprotonation of the radical benzothiophene precursors are accomplished by a [Co^III^] catalyst in a second catalytic cycle. [Co^III^] is stepwise reduced to [Co^I^]. Protonation leads to a [Co^III^] hydride complex generating dihydrogen by protonation. Quantitative H_2_ production could only be achieved by careful selection and adjustment of the type and amount of basic additives. The scope of this method includes electron-rich and electron-deficient 2-aryl-substituted benzothiophenes. However, strongly electron-donating methoxy or electron-withdrawing trifluoromethyl groups decreased the yield of the reaction. A series of 2-alkylsubstituted benzothiophenes were prepared and the method was applied for the synthesis of antitumor agents.

**Scheme 33:**
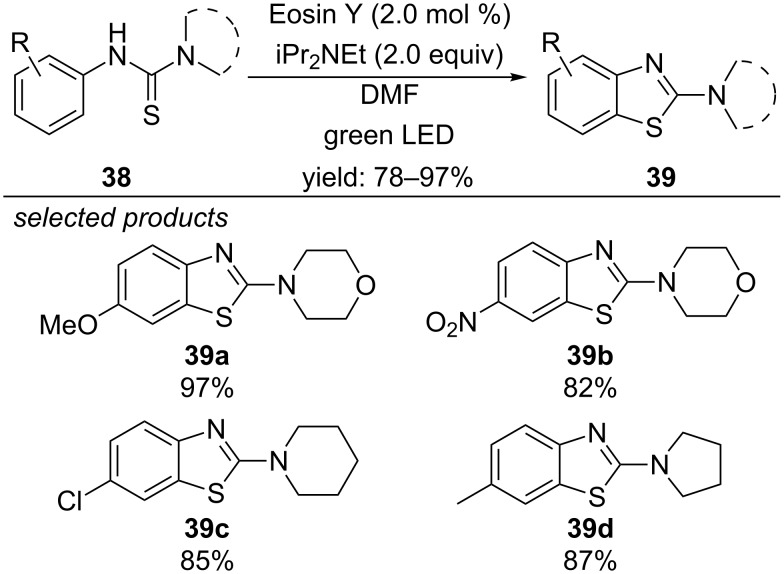
Metal-free photocatalyzed synthesis of 2-aminobenzothiazoles, applying Eosin Y as photocatalyst.

In the same year, the group of Singh published two similar, but metal-free procedures. In the first report a new photocatalyzed procedure for the preparation of 2-aminobenzothioazoles from the respective *N*-aryl thioureas with Eosin Y as photoredox catalyst and molecular oxygen as terminal oxidant was shown (Scheme 33) [68]. They propose a mechanism where the excited state of Eosin Y is quenched reductively by the deprotonated thiourea derivative giving the Eosin Y radical anion and a thiyl radical. Aerobic oxidation closes the catalytic cycle and after hydrogen atom abstraction of the carbon-centred radical intermediate the final product is formed.

#### Formation of 1,3,4-thiadiazoles

**Scheme 34:**
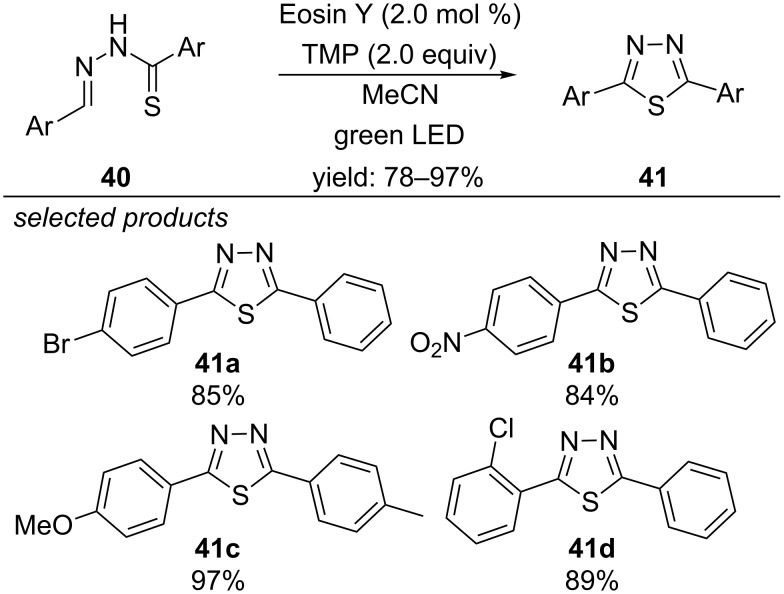
Metal-free photocatalyzed synthesis of 1,3,4-thiadiazoles, using Eosin Y as photocatalyst.

In a second article the same concept led to the formation of 1,3,4-thiadiazoles (Scheme 34) [69]. Again, the sulfur anion is photooxidized by Eosin Y to produce a thiyl radical intermediate. Aerobic oxidation regenerates the photocatalyst and forms a superoxide radical anion. Subsequent thiyl radical addition to the imine moiety forms the five-membered ring, which is aromatized by hydrogen atom abstraction with the superoxide radical.

### Sulfides

#### Formation of benzothiophenes

**Scheme 35:**
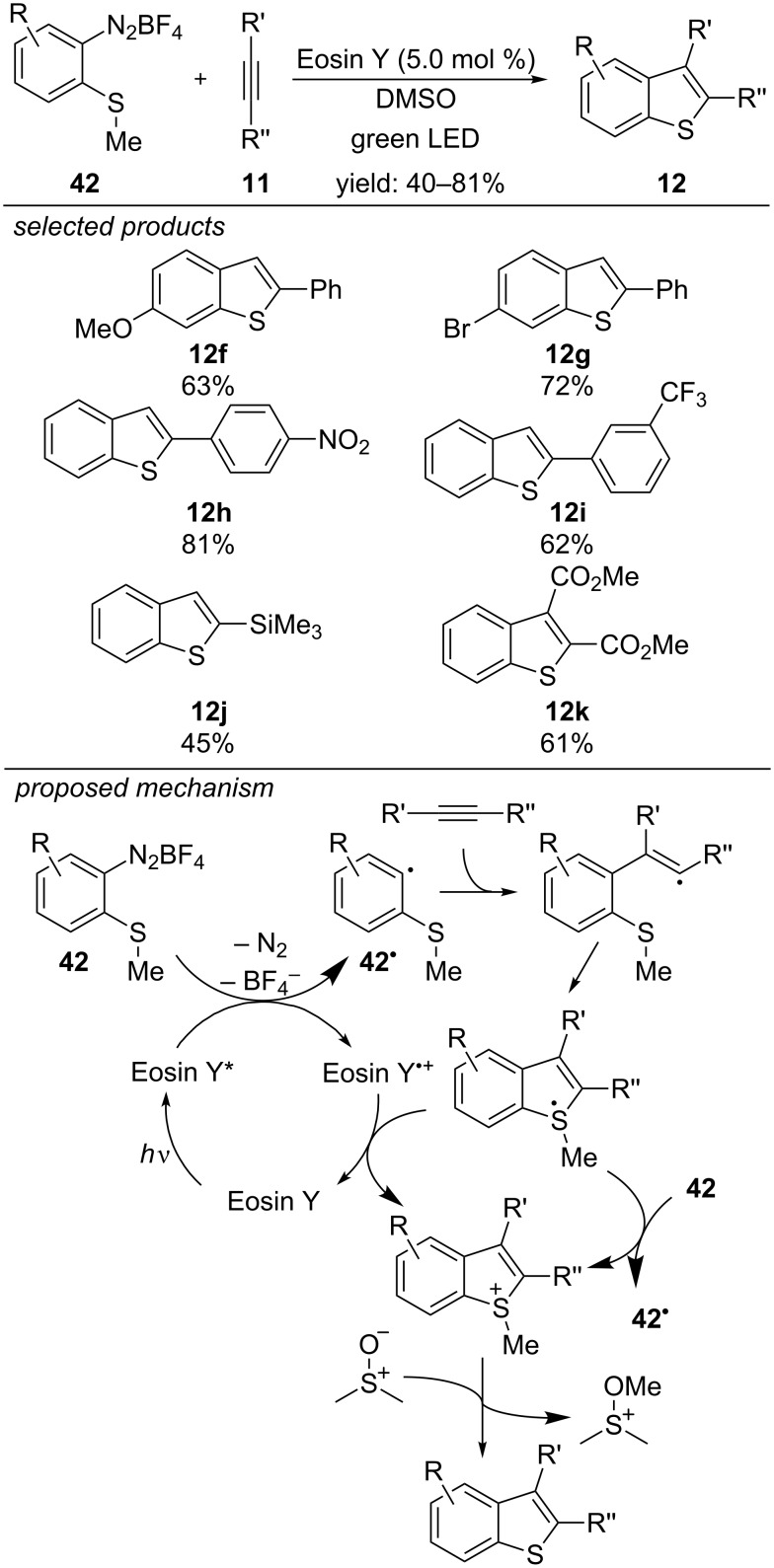
Visible-light photoredox-catalyzed preparation of benzothiophenes with Eosin Y.

A metal-free visible-light photoredox-catalyzed approach for the rapid synthesis of 2-substituted benzothiophenes applying Eosin Y as photocatalyst was reported by our laboratory (Scheme 35) [70]. *o*-Methylthioaryl diazonium salts and phenylacetylene are starting materials, and photoexcited Eosin Y transfers an electron to the diazonium salt, which decomposes into a reactive aryl radical and N_2_. Addition to the acetylene yields a vinylic radical intermediate, which cyclizes to the respective sulfuranyl radical. Subsequent oxidation closes the catalytic cycle and generates a sulfur-centred cation. Finally, demethylation affords the desired substituted benzothiophene.

**Scheme 36:**
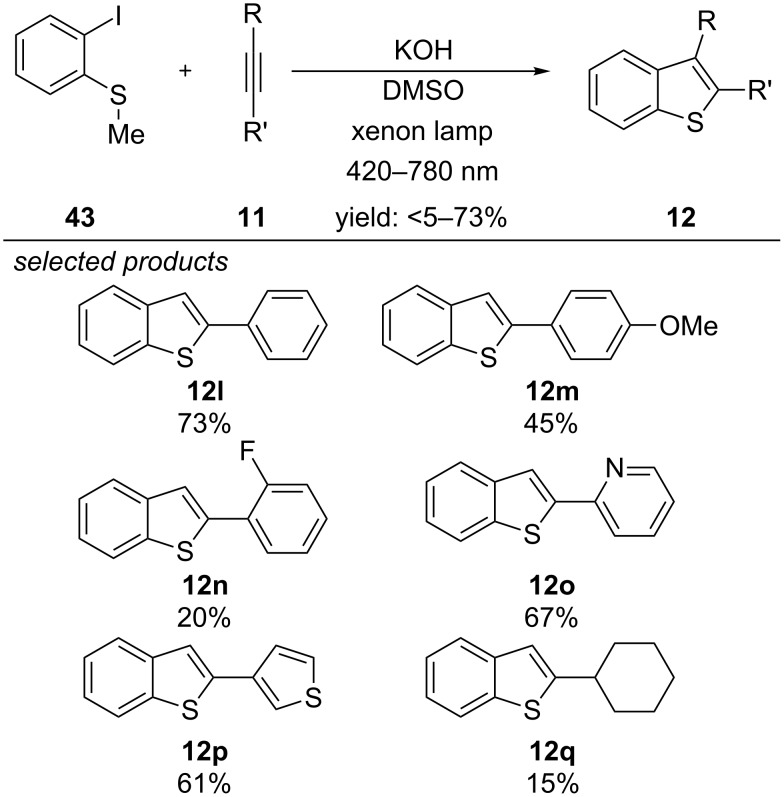
Visible-light-induced KOH/DMSO superbase-promoted preparation of benzothiophenes.

In 2016, Yuan and co-workers developed a different synthesis of benzothiophenes (Scheme 36) [71]. They found, that a combination of KOH/DMSO and 2-halothioanisole forms an orange adduct, which absorbs visible-light. They propose that KOH/DMSO as a superbase can photoreduce the aryl halide, which subsequently generates an aryl radical by expelling the halide anion. Radical addition to the acetylene derivative yields a vinyl radical, which cyclizes to the respective sulfuranyl radical. Oxidation of this intermediate by the radical cation of the superbase closes the electron transfer cycle. Rearomatization of the benzothiophene proceeds via demethylation, accomplished by a hydroxide anion. They were able to perform the reaction with different electron-rich, halogenated or heterocycle substituted alkynes, affording the respective products in good to moderate yields. Strongly electron-withdrawing nitro, acyl or cyano groups were not suitable. In accordance to the proposed mechanism, the reaction with 2-iodothioanisoles gave better yields than their bromo or chloroanalogues, because of their higher fragmentation stability after single-electron reduction.

### Disulfides

#### Formation of sulfides

**Scheme 37:**
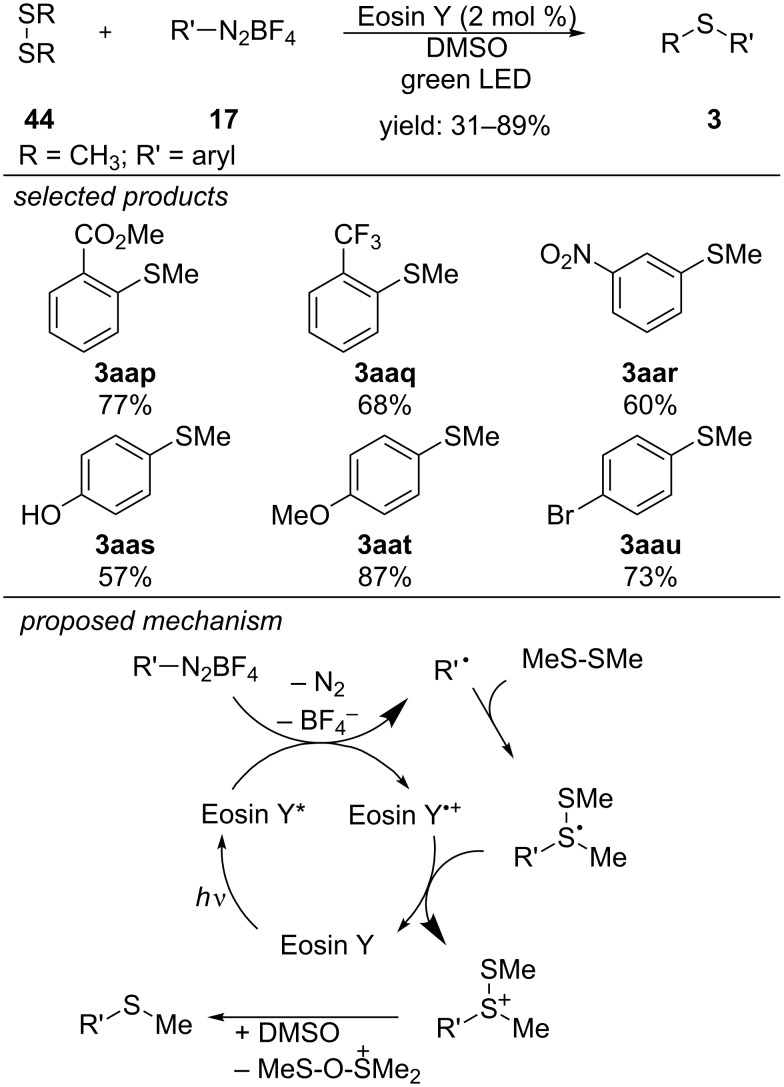
Jacobi von Wangelin’s photocatalytic approach for the synthesis of aryl sulfides, applying Eosin Y as photocatalyst.

Jacobi von Wangelin and co-workers published a photocatalyzed protocol for the formation of aryl sulfides from the respective aryl diazonium salts and dimethyl disulfide (Scheme 37) [72]. They applied Eosin Y for the photoreduction of aryl diazonium salts, in order to generate the respective aryl radicals. Radical addition to the nucleophilic disulfide forms a stabilized trivalent sulfur-centred radical. Oxidation of this intermediate by the Eosin Y radical cation closes the catalytic cycle and the sulfur-centred cationic species is formed. The desired aryl sulfide is obtained by S–S bond cleavage. Several substituted aryl diazonium salts could be applied for the reaction with dimethyl disulfide, including esters, nitro, hydroxy, trifluoromethyl groups and halides, but iodides led to polymer formation.

**Scheme 38:**
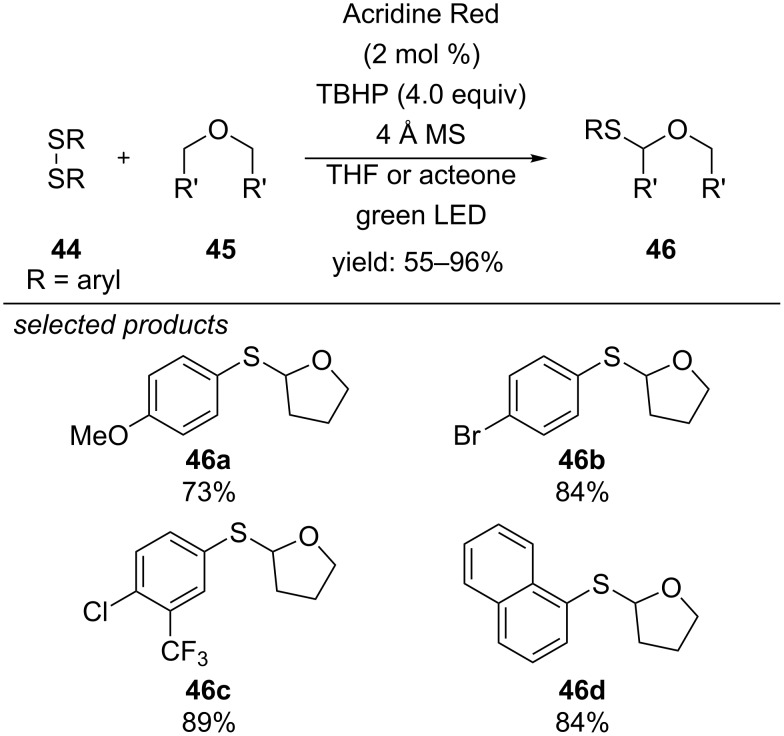
Visible-light photosensitized α-C(sp^3^)–H thiolation of aliphatic ethers.

Li and Wang developed a method for the α-C(sp^3^)–H thiolation of ethers, using Acridine Red as photosensitizer and *tert*-butyl hydroperoxide (TBHP) as oxidant (Scheme 38) [73]. They reported that photoexcited Acridine Red performs an energy transfer on TBHP, which homolytically decomposes to generate a hydroxyl radical and a *tert*-butoxyl radical. Both species are able to abstract a hydrogen atom from the α-position of the ether. The obtained carbon-centred radical can further react with the nucleophilic disulfide to form the respective thiolated ether adduct. A new sulfenyl radical is generated in the last step and reacts with another alkoxyalkyl radical. A series of functionalized diaryl disulfides, containing electron-donating and withdrawing-groups and also sterically demanding substituents gave the respective products. Dialkyl disulfides did not react under the reported conditions. However, cyclic tetrahydrofurans and linear aliphatic ethers are suitable substrates for the reaction.

### Organo thiosulfates

#### Formation of sulfides

**Scheme 39:**
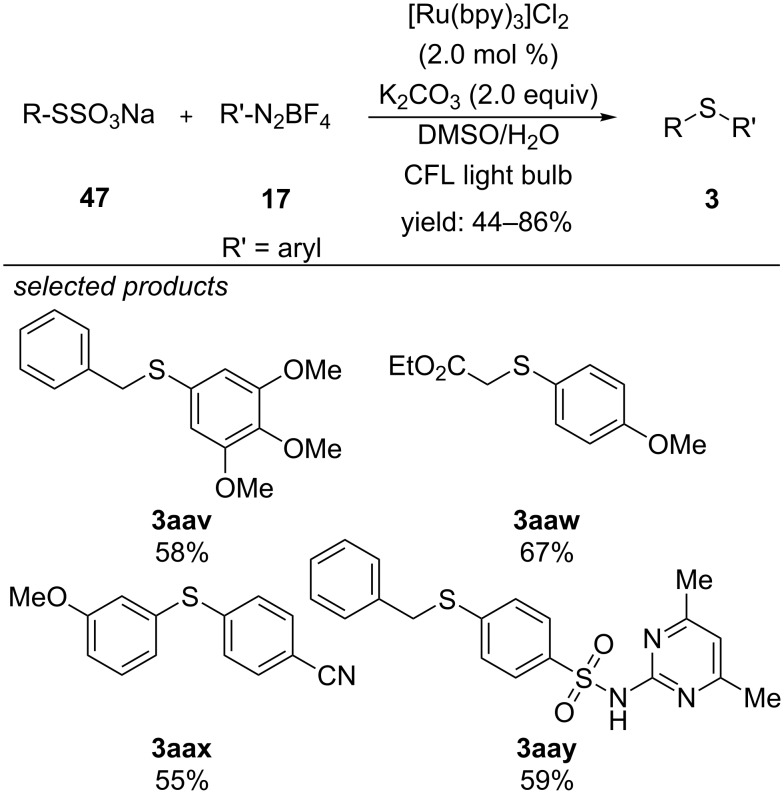
Visible-light photocatalyzed cross-coupling of alkyl and aryl thiosulfates with aryl diazonium salts, applying [Ru(bpy)_3_]Cl_2_.

In 2015, Jiang and co-workers reported a visible-light photocatalyzed method for the preparation of sulfides from alkyl and aryl thiosulfates and aryl diazonium salts (Scheme 39) [74]. They confirmed by transient absorption spectroscopy that a single-electron transfer occurs between [Ru(bpy)_3_]Cl_2_ and the aryl diazonium salt. Additionally, electron paramagnetic resonance studies showed that K_2_CO_3_ interacts with the thiosulfate and facilitates the formation of radical species, which leads to higher product formation. Alkyl and aryl thiosulfates reacted with a series of substituted aryl- and heteroaryl diazonium salts in high yields. In addition, complex aryl diazonium salts were successfully coupled to the corresponding sulfides.

**Scheme 40:**
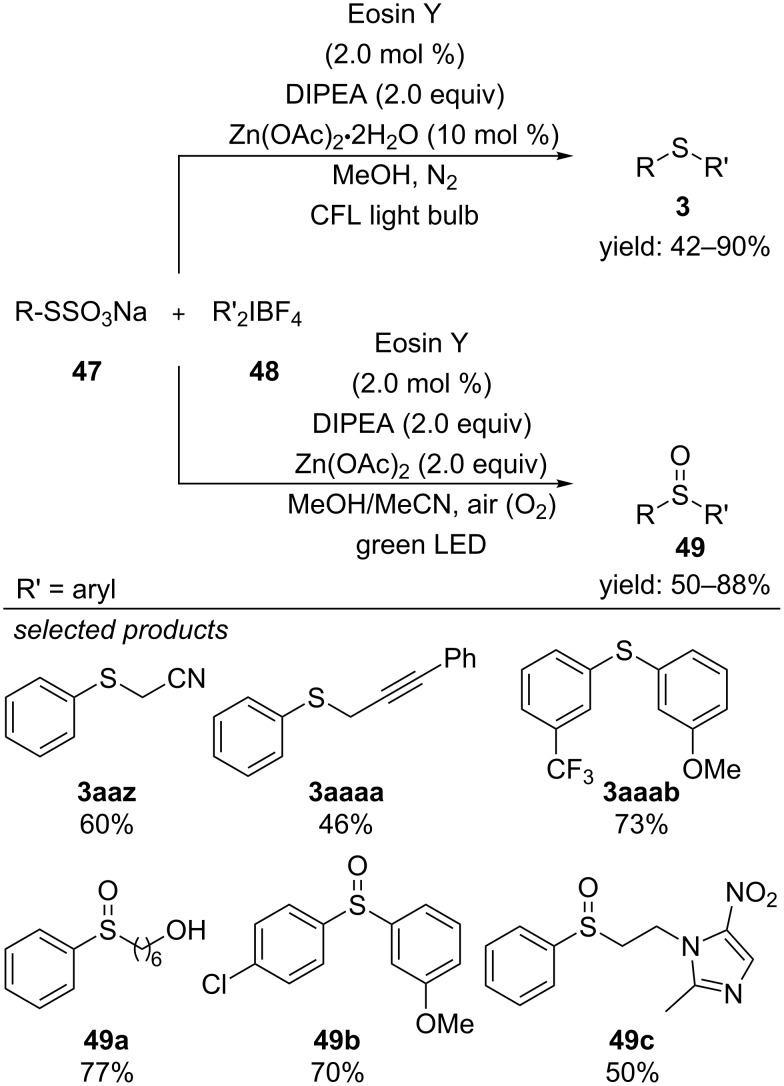
Visible-light photocatalyzed, controllable sulfenylation and sulfoxidation with organic thiosulfate salts.

Very recently, the same group reported of a controllable sulfenylation and sulfoxidation procedure starting from alkyl and aryl thiosulfates and diaryliodonium salts under visible-light catalysis with Eosin Y (Scheme 40) [75]. They observed that the reaction yields the respective sulfides when it is conducted under inert atmosphere, whereas aerobic conditions selectively lead to the respective sulfoxides. They propose a mechanism where first the diaryliodonium salt is photoreduced by the excited-state of Eosin Y, yielding an aryl radical and Eosin Y radical cation, respectively. Radical cross-coupling with the thiosulfate salt forms a sulfide radical anion, which is oxidized to the respective sulfide adduct by regeneration Eosin Y. This sulfide-forming step counts also for the sulfoxidation reaction. However, selective oxidation of the sulfide to the respective sulfoxide is accomplished by in situ generated singlet oxygen, which is generated by energy-transfer of the excited state of Eosin Y and only possible under aerobic conditions. The authors also found that zinc acetate is beneficial for the selective oxidation. A series of electron-rich and electron-poor diaryliodonium salts reacted efficiently with alkyl and aryl thiosulfate salts to the respective sulfide and sulfoxides. A row of functional groups like for example amines, cyano groups, esters, hydroxy groups or nitro groups were highly tolerated and gave the opportunity for late-stage functionalization of complex molecules.

### Dimethyl sulfoxide

#### Formation of aryl methyl sulfoxides

**Scheme 41:**
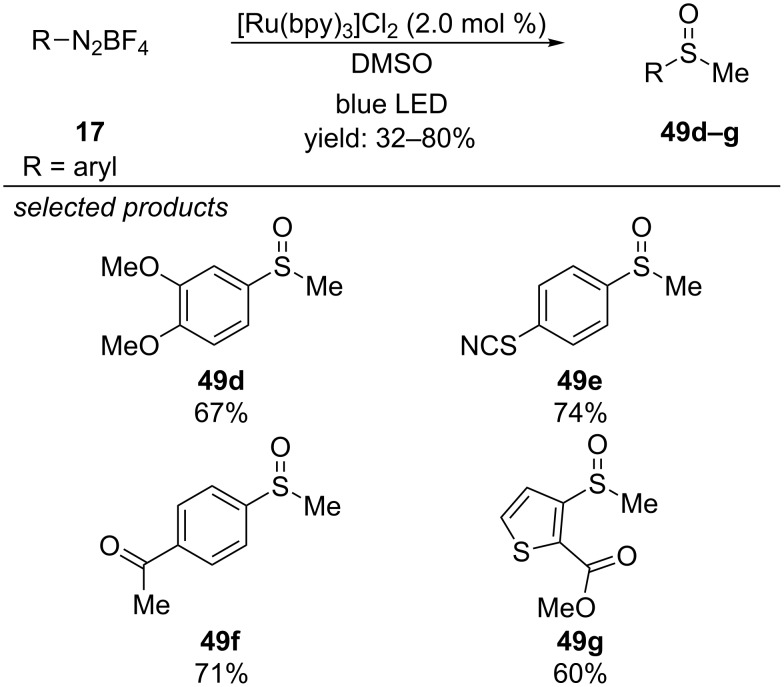
Rastogi’s photoredox-catalyzed methylsulfoxidation of aryl diazonium salts, using [Ru(bpy)_3_]Cl_2_ as photocatalyst.

Rastogi and co-workers reported the visible-light photoredox-catalyzed methylsulfoxidation reaction of aryl diazonium salts with [Ru(bpy)_3_]Cl_2_ as photocatalyst (Scheme 41) [76]. The authors propose that photoexcited [Ru(bpy)_3_]^2+^* is oxidatively quenched by the aryl diazonium salt, generating [Ru(bpy)_3_]^3+^ and an aryl radical intermediate. The radical addition to DMSO and subsequent single-electron oxidation, either by regenerating the photocatalyst or by forming a new aryl radical from the aryl diazonium salt, leads to a sulfur-centred cation. Methyl-transfer affords the desired aryl methyl sulfoxide. The scope of this reaction included various electron-rich and poor aryl and heteroaryl diazonium salts. Diverse functional groups, like nitriles, thiocyanates ketones or esters were tolerated.

### Sulfinic acids and sulfinate salts

#### Formation of allyl and vinyl sulfones

**Scheme 42:**
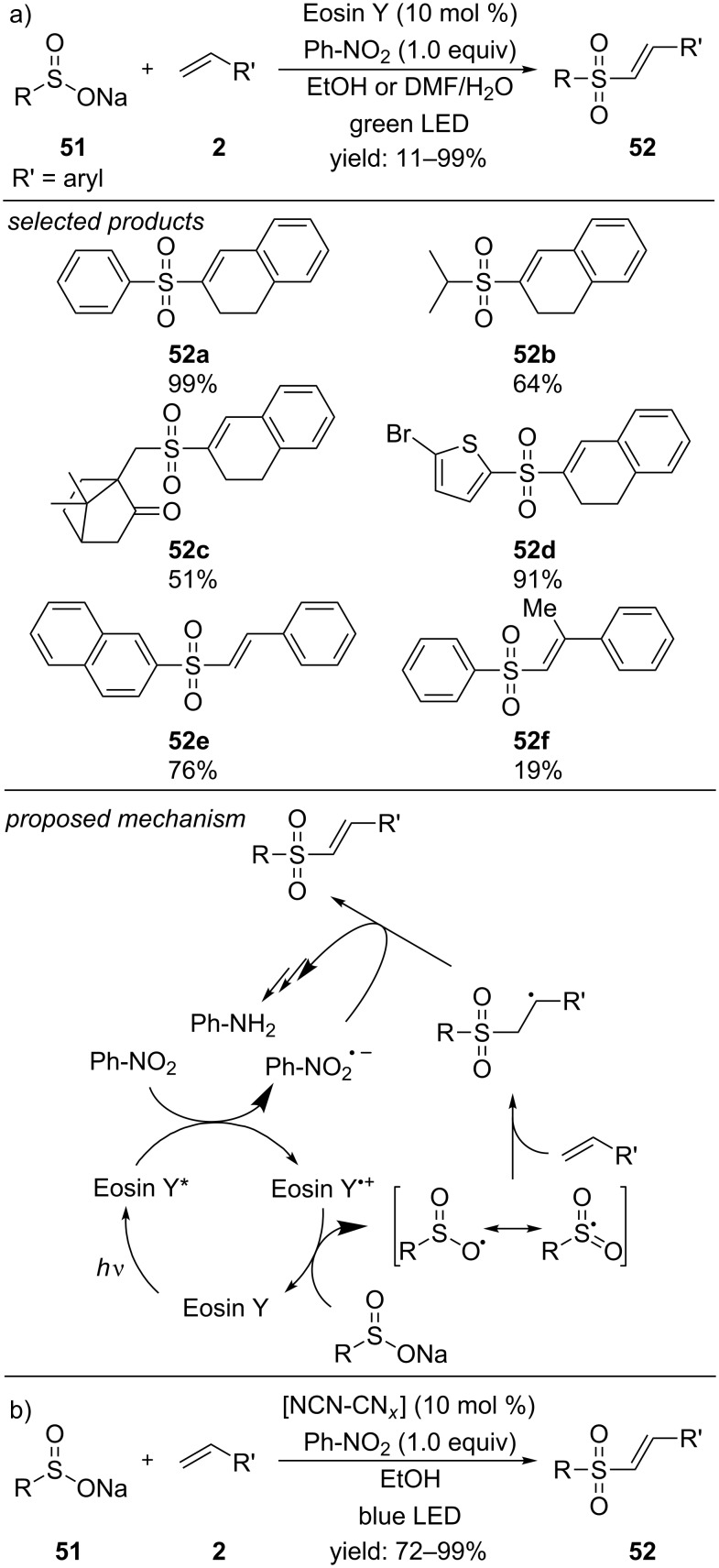
a) Visible-light metal-free Eosin Y-catalyzed procedure for the preparation of vinyl sulfones from alkyl and (hetero)aryl sulfinate salts. b) Visible-light catalyzed preparation of vinyl sulfones, applying heterogeneous carbon nitride photocatalysts.

In 2015, our laboratory reported the first metal-free visible-light photoredox-catalyzed method for the preparation of vinyl sulfones from the respective aryl sulfinate salts (Scheme 42a) [77]. Eosin Y was applied as photocatalyst to produce a diverse scope of vinyl sulfones. For styrene derivatives, the solvent had to be changed from ethanol to a mixture of DMF/H_2_O as the nucleophile ethanol leads to a byproduct formation. The reaction proceeds via oxidative quenching of the photoexcited state of Eosin Y by nitrobenzene to generate the Eosin Y radical cation. In order to close the catalytic cycle, the aryl sulfinate salt is oxidized to the respective sulfur-centred radical, which adds regioselectively to the alkene moiety. Further oxidation and deprotonation leads to the respective vinyl sulfone. One year later, we successfully expanded the scope of the Eosin Y-catalyzed synthesis of vinyl sulfones. In addition to aryl sulfinate salts, also alkyl and heteroaryl sulfinate salts were applied in the reported method (Scheme 42a) [78]. Short aliphatic alkyl chains as well as sterically demanding cyclohexyl or 10-camphor sulfinate salts and substituted 2-thiophene sulfinate salts afforded moderate to high yields of the respective products. In this study, we could also exclude a reductive quenching cycle by transient spectroscopy. Very recently a heterogeneous modified carbon nitride photocatalyst could successfully be applied to the synthesis of vinyl sulfones (Scheme 42b) [79]. The catalyst was recycled and reused after centrifugation and washing with ethanol. High catalytic activity was preserved for at least 3 cycles.

**Scheme 43:**
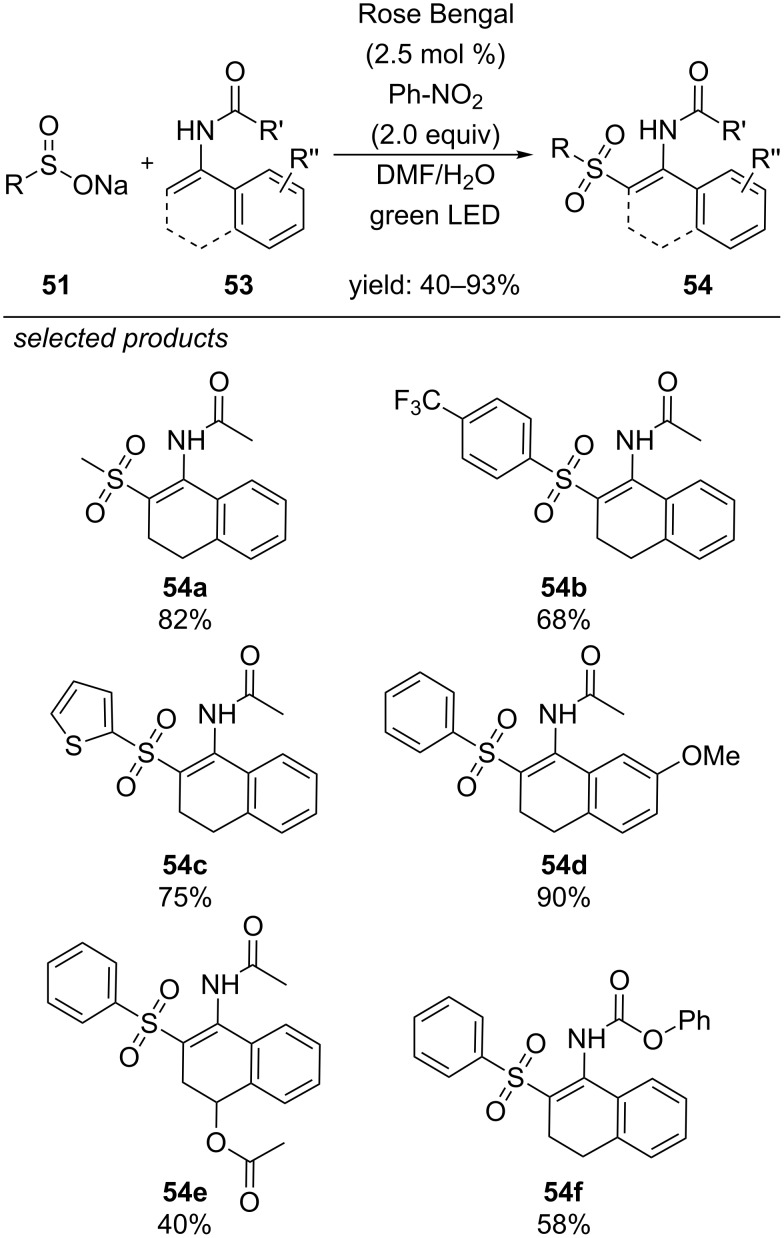
Visible-light photocatalyzed cross-coupling of sodium sulfinates with secondary enamides.

Very recently, the group of Zhang published a photocatalyzed procedure for the preparation of β-acetylaminoacrylsulfones from the respective sodium sulfinates and enamides, applying Rose Bengal as photocatalyst and nitrobenzene as terminal oxidant (Scheme 43) [80]. In general, this procedure is suitable for the cross-coupling of a variety of substituted secondary enamides with alkyl, aryl and heteroaryl sodium sulfinate salts. Tertiary enamides could not be reacted with sodium sulfinates to yield the respective products.

**Scheme 44:**
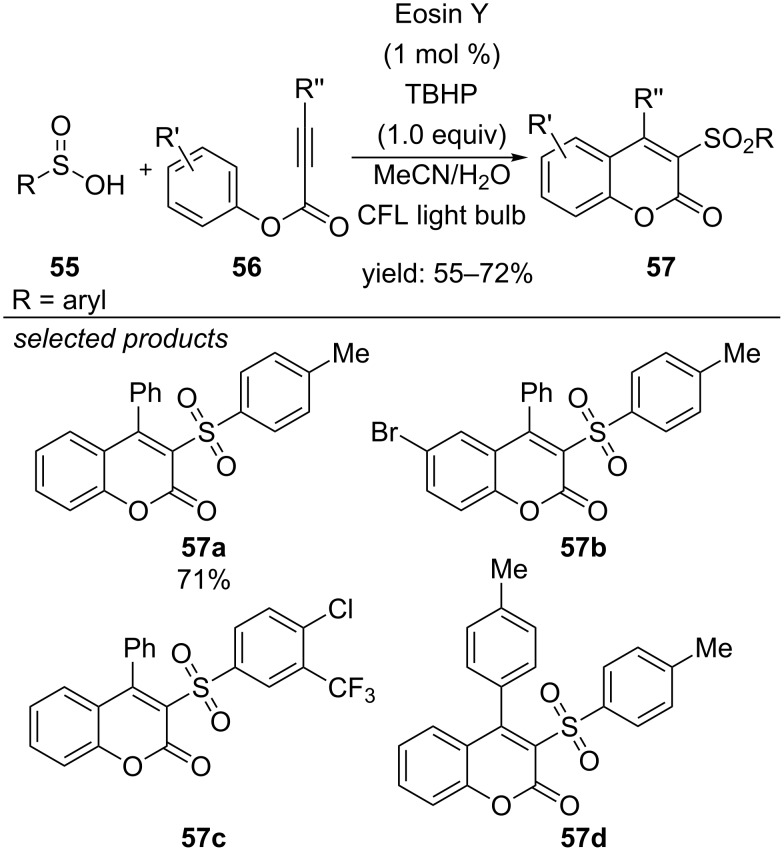
Wang’s photocatalyzed oxidative cyclization of phenyl propiolates with sulfinic acids, applying Eosin Y as organic dye.

Wang and co-workers described a new method for the cyclization of phenyl propiolates with sulfinic acids, generating valuable coumarin derivatives in 2015 (Scheme 44) [81]. Visible-light irradiation of Eosin Y allows the reduction of *tert*-butyl hydroperoxide (TBHP) to form a reactive *tert*-butoxyl radical. After hydrogen abstraction from the sulfinic acid and subsequent radical addition to the alkyne derivative, consecutive steps for rearomatization lead to the cyclic coumarin adduct. The authors used various electron-donating alkyl-substituted and halogenated phenyl propiolates in the reaction. They found that *para*- and *meta*-substituted derivatives cyclize in moderate to good yields, whereas substituents in *ortho*-position were sterically too demanding and only traces of the desired products could be obtained.

**Scheme 45:**
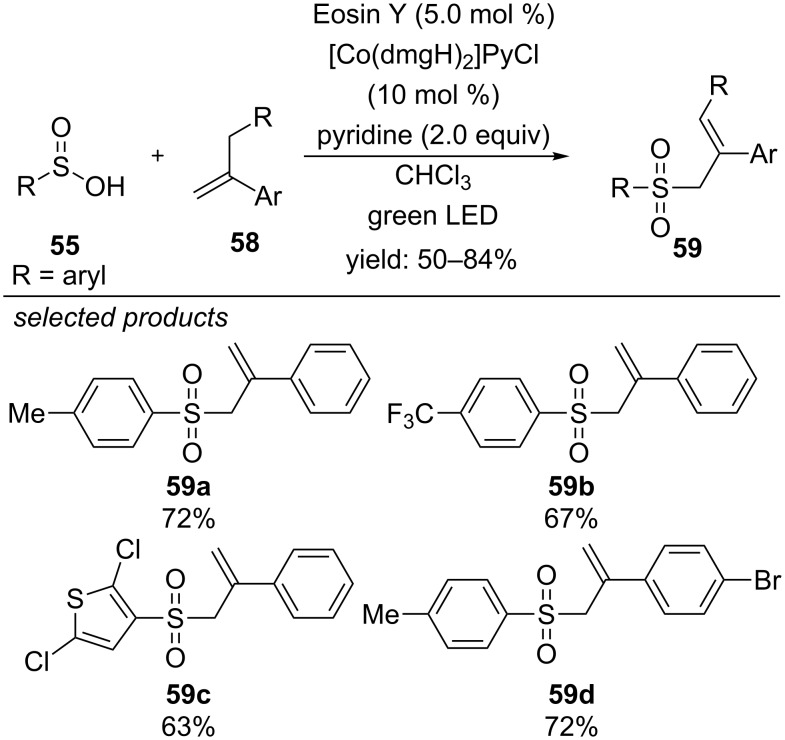
Lei’s sacrificial oxidant-free synthesis of allyl sulfones by merging photoredox and transition metal catalysis.

One year later, Lei and co-workers reported a photocatalyzed oxidant-free method for the preparation of allyl sulfones from sulfinic acids and α-methylsytrenes, applying Eosin Y as organic photocatalyst and avoiding any sacrificial additives or oxidants (Scheme 45) [82]. They successfully applied the proton reducing [Co^III^] catalyst to form H_2_ as the only byproduct. The reaction was suitable for various substituted sulfinic acids and α-methylsytrenes. Electron-donating methyl or methoxy substituents, as well as electron-withdrawing halogen or trifluoromethyl groups were tolerated for both, sulfinic acids and styrenes. Even aliphatic methane sulfinic acid could be converted to the respective allyl sulfone in moderate yield. The authors propose a reductive photocatalytic cycle, where the aryl sulfinic acid anion is photooxidized by the excited state of Eosin Y. The sulfur-centred radical adds to the alkene to generate a carbon-centred radical intermediate. The cobalt-catalyst is involved in two ways: oxidation and deprotonation of the radical species yielding the desired product and oxidation of the Eosin Y radical anion to close the photocatalytic cycle. The H_2_ evolution was not quantitative, which was explained by the decomposition of the organic photocatalyst during the H_2_ evolution process. Increasing amounts of the photocatalyst increased the yield of product and H_2._

**Scheme 46:**
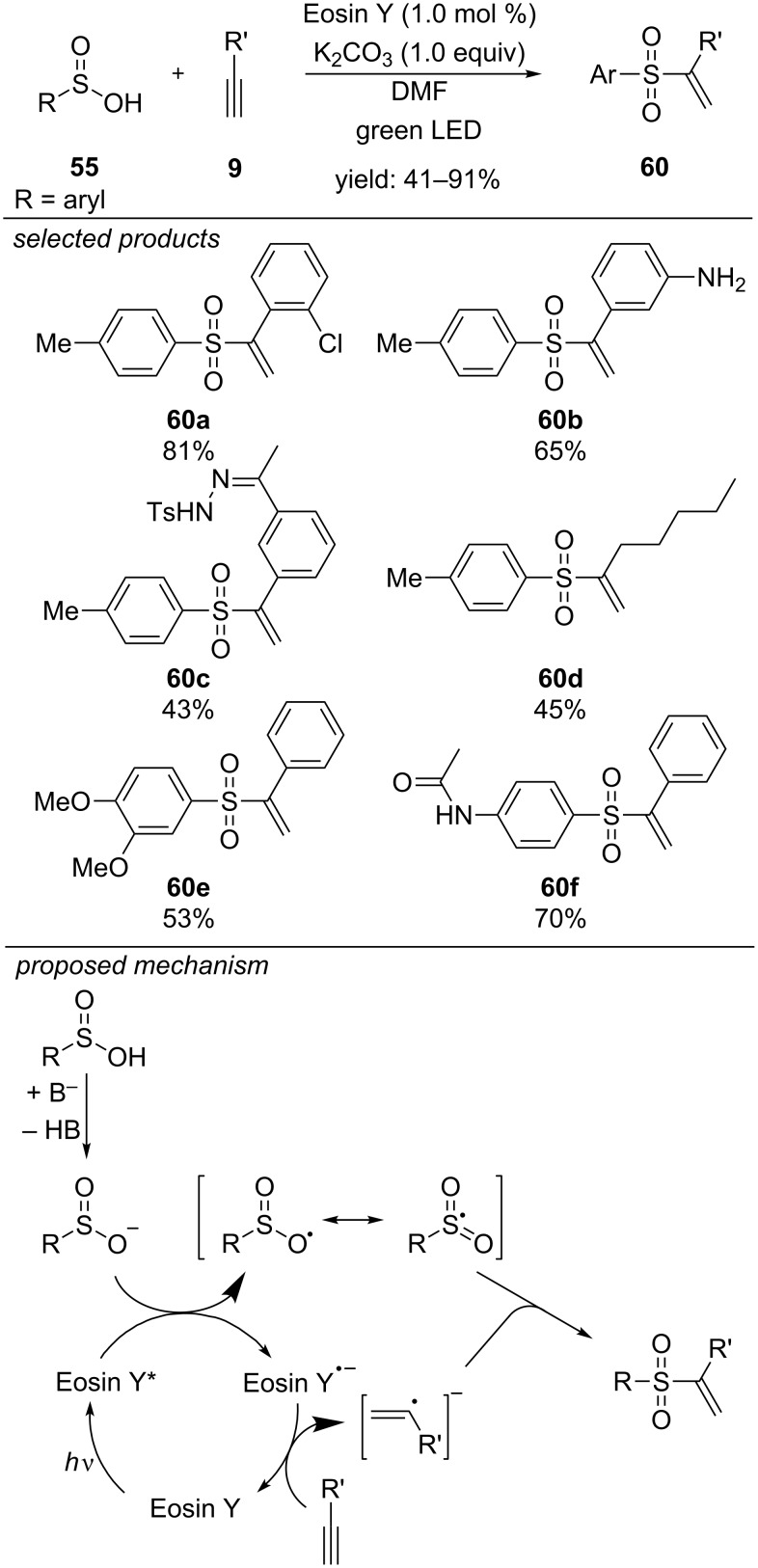
Photocatalyzed Markovnikov-selective radical/radical cross-coupling of aryl sulfinic acids and terminal alkynes.

Very recently, the same group reported a regioselective photoredox-catalyzed preparation of vinyl sulfones in Markovnikov orientation (Scheme 46) [83]. By using Eosin Y as organic dye, aryl sulfinic acids could efficiently be cross-coupled with terminal alkynes. The Markovnikov regioselectivity is rationalized by a radical/radical cross-coupling pathway, instead of radical addition to an unsaturated system. The authors propose that Eosin Y as photoredox catalyst generates both, the sulfur-centred radical of the sulfinic acid as well as the α-vinyl carbon-centred radical. Radical/radical cross-coupling leads to the desired vinyl sulfone in Markovnikov orientation. The reaction is applicable to diverse functionalized terminal alkynes, bearing electron-donating (methyl, methoxy, amine or hydroxy) and withdrawing substituents (halides, carbonyls or sulfonamides) and also heterocyclic substrates. Various aromatic sulfinic acids could be applied, including methoxy, halogen, trifluoromethyl or acetylamino-substituted benzenes, as well as one thiophene derivative.

### Formation of other sulfone derivatives

**Scheme 47:**
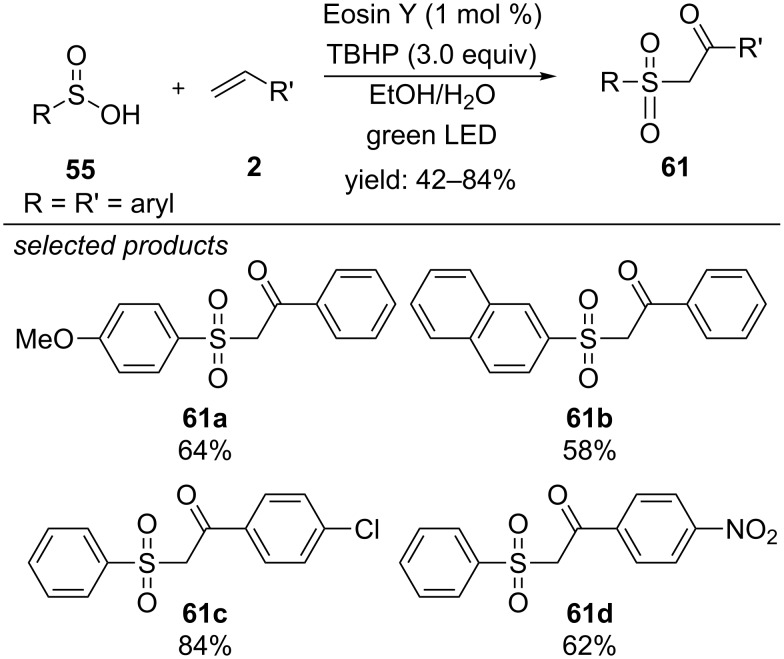
Visible-light Eosin Y induced cross-coupling of aryl sulfinic acids and styrene derivatives, affording β-ketosulfones.

The groups of Yang and Wang reported in 2016, that β-ketosulfones can be obtained by direct visible-light induced oxysulfonylation of styrenes, applying similar photoredox conditions as reported by Wang and co-workers [81] one year before (Scheme 47) [84]. They propose that either a hydroxide anion, derived from cleaved TBHP, or a water molecule (solvent) attacks nucleophilic on the cationic intermediate in anti-Markovnikov orientation. The resulting β-hydroxysulfone is then further oxidized to the respective β-ketosulfone. Various styrene derivatives were converted, but aliphatic alkenes did not react. Functionalities such as methyl, nitro or cyano groups and halogen substituents are tolerated. The aryl sulfinic acids allow electron-donating groups.

**Scheme 48:**
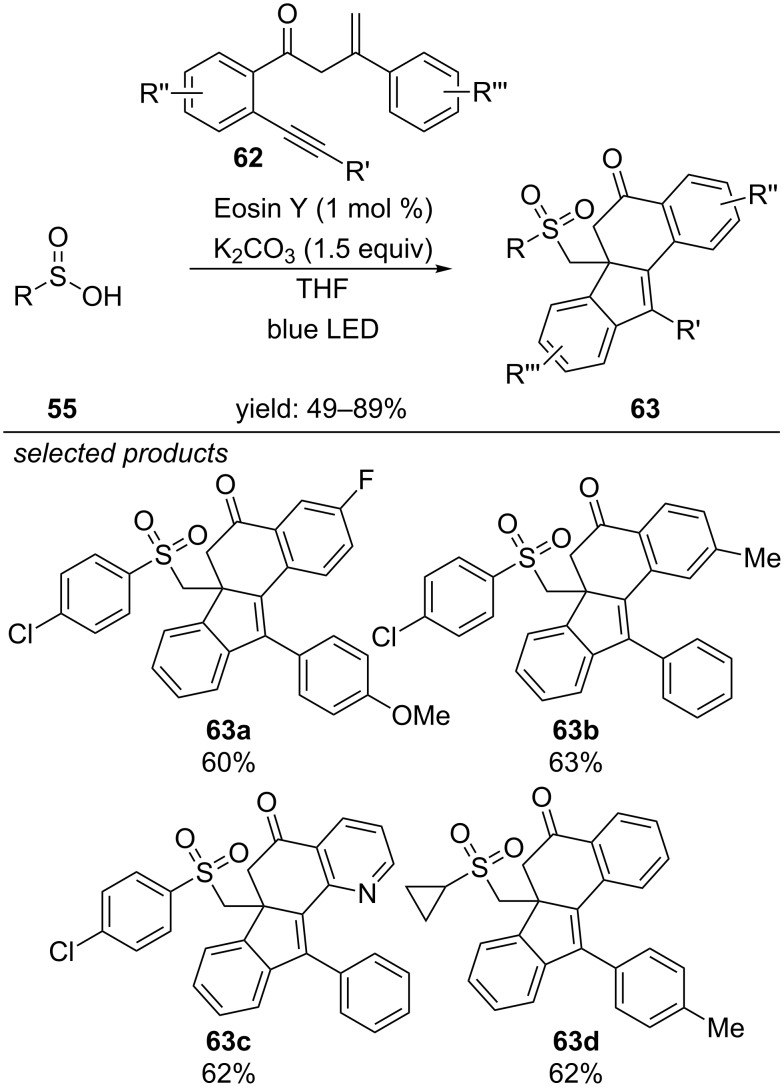
Photoredox-catalyzed bicyclization of 1,7-enynes with sulfinic acids, applying Eosin Y as photocatalyst.

Very recently Wang and Jiang reported the Eosin Y-photocatalyzed bicyclization of 1,7-enynes, forming sulfonylated benzo[α]fluoren-5-ones (Scheme 48) [85]. The reaction requires argon atmosphere, as the yield decreased under aerobic conditions. They propose a mechanism, which proceeds via a reductive quenching cycle of Eosin Y. After the addition of the sulfonyl radical to the alkene moiety of the 1,7-enyne, two consecutive cyclizations lead to the final sulfonylated benzo[α]fluoren-5-one. Electron and proton transfer and subsequent formation of dihydrogen close the catalytic cycle and regenerate the photocatalyst.

### Sulfinamides

#### Formation of sulfoxides

**Scheme 49:**
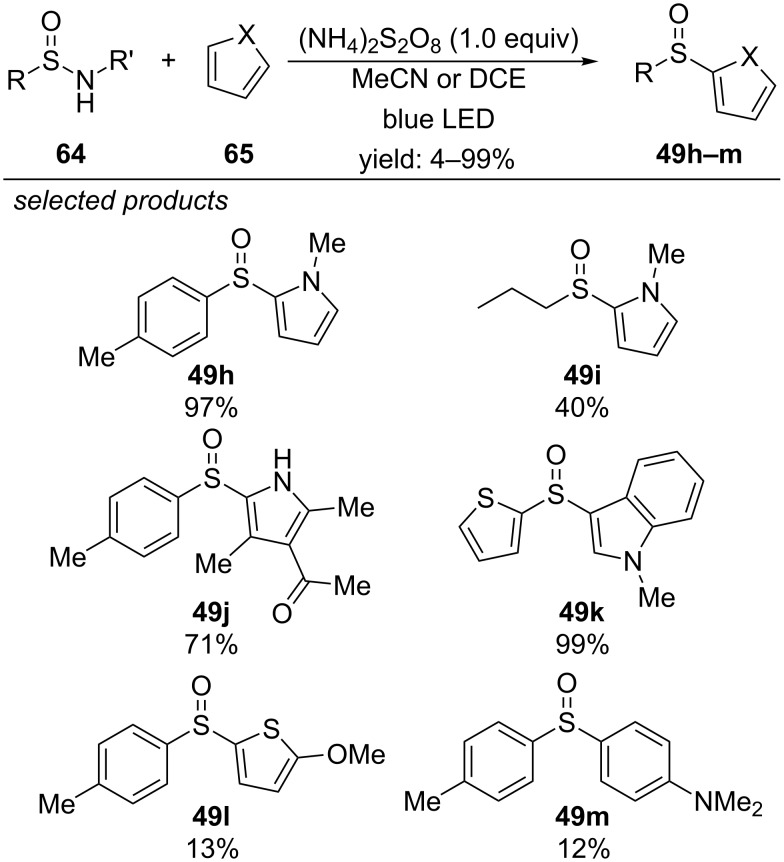
Visible-light-accelerated C–H-sulfinylation of arenes and heteroarenes.

A new method for the preparation of sulfoxides was recently reported by our laboratory (Scheme 49) [86]. Sulfinamides were applied as sulfinylation reagents for a diversified scope of arenes and heteroarenes with ammonium persulfate ((NH_4_)_2_S_2_O_8_) as oxidant under irradiation with visible light. Although none of the starting materials absorbs visible-light, the mixture of all reagents shows absorption in the visible-light region, which indicates the formation of donor–acceptor complexes. The exact origin of the absorption could not be specified until now. The reaction tolerates diverse functional groups. Electron-rich arenes react well and substituted pyrroles and indoles give the corresponding sulfoxides in high yields. Less electron-rich thiophene or benzene derivatives gave low yields. Nevertheless, carbocyclic azulene afforded the respective sulfoxide in 88% yield. We propose an electrophilic aromatic substitution mechanism, where the sulfinamide is oxidized by ammonium persulfate to the respective sulfur-centred cationic intermediate. After electrophilic aromatic substitution, the amine moiety is cleaved and the corresponding sulfoxide is formed. The mechanistic proposal is supported by competition experiments using two arenes with different nucleophilicity, which were reacted with one sulfinamide. The respective product with the stronger nucleophile is formed exclusively. In reactions with two arenes having similar nucleophilicity a mixture of the respective products was obtained.

### Sulfonyl halides and selenosulfonates

#### Formation of sulfone derivatives

**Scheme 50:**
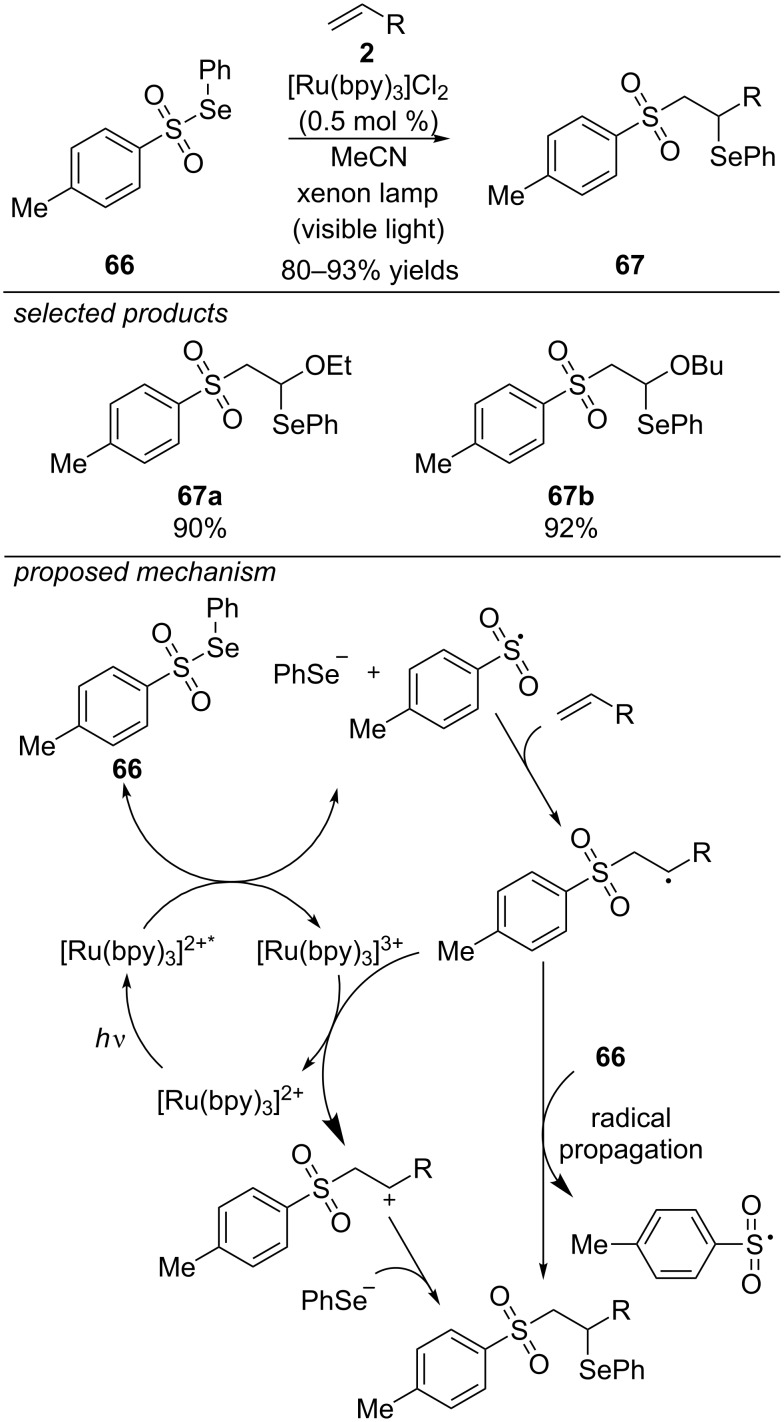
Visible-light photoredox-catalyzed β-selenosulfonylation of electron-rich olefins, applying [Ru(bpy)_3_]Cl_2_ as photocatalyst.

Already in 1994, the group of Barton envisioned that the photoreduction of selenosulfonates by [Ru(bpy)_3_]Cl_2_ as photoredox catalyst could lead to reactive sulfonyl radicals (Scheme 50) [87]. They were the first to report on a visible-light induced sulfonylation of olefins. For their reaction, they applied *p*-toluene selenosulfonate and found that especially electron-rich olefins worked well under their conditions. The difunctionalized products (β-selenosulfones) were obtained in high yields from a series of alkyl vinyl ethers. The reaction proceeds via an oxidative photocatalytic cycle. The addition of the sulfonyl radical to the olefin yields a β-sulfonyl radical intermediate. The final difunctionalized product can be generated by two possible pathways: Either the radical intermediate is oxidized to the respective cation by closing the catalytic cycle and combines with a selanolate anion. Alternatively, propagation of the radical chain forms the desired product and a new equivalent of the sulfonyl radical.

**Scheme 51:**
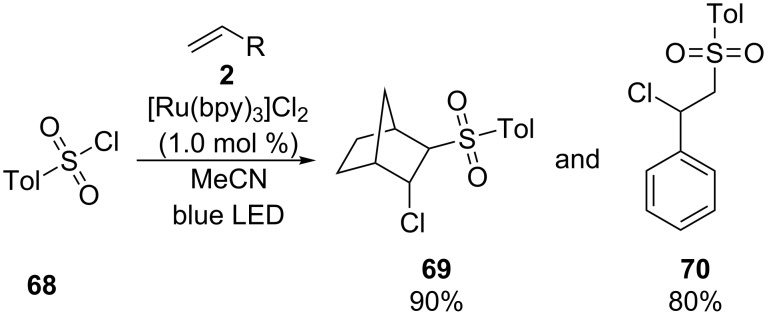
Photocatalyzed preparation of β-chlorosulfones from the respective olefins and *p*-toluenesulfonyl chloride, using [Ru(bpy)_3_]Cl_2_ as photocatalyst.

Stephenson and co-workers performed a similar reaction in 2012, where they applied *p*-toluenesulfonyl chloride in the corresponding β-chlorosulfonylation (Scheme 51) [88]. Two examples, norbonene and styrene, were presented and [Ru(bpy)_3_]Cl_2_ was used as photoredox catalyst.

**Scheme 52:**
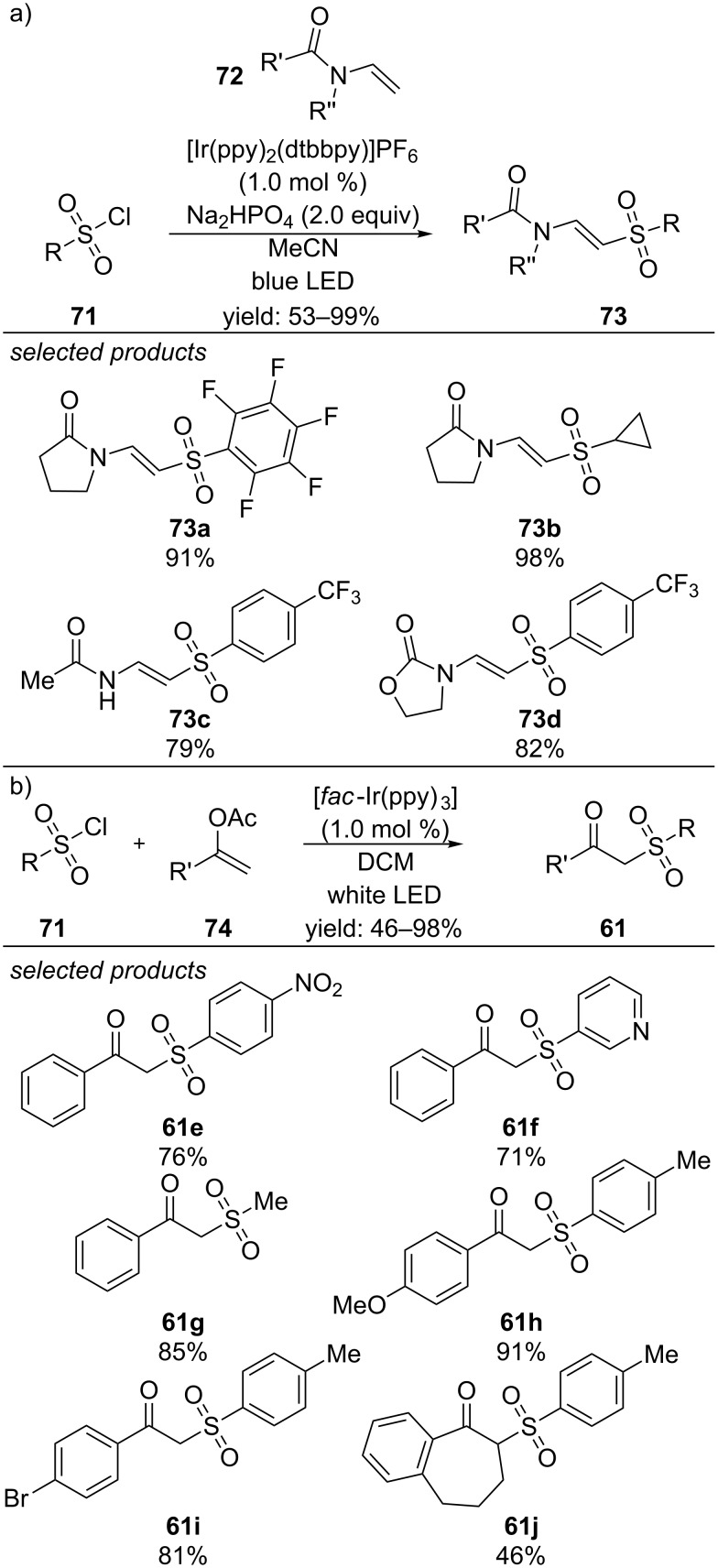
a) Photocatalyzed preparation of β-amidovinyl sulfones from sulfonyl chlorides. b) Preparation of β-ketosulfones by photoredox-catalyzed coupling of sulfonyl chlorides with enol acetates.

The groups of Zhang and Yu reported two additional photoredox-catalyzed methods for the sulfonylation of olefins. In their first paper they applied enamides as olefins, which were sulfonylated to the respective β-amidovinyl sulfones under irradiation with visible light and [Ir(ppy)_2_(dtbbpy)]PF_6_ as photocatalyst (Scheme 52a) [89]. The scope of this method includes cross-coupling of electron-rich, neutral and electron-deficient (hetero)aromaticsulfonyl chlorides and even alkylsulfonyl chlorides with (a) cyclic enamides, different *N*-vinyllactams and enecarbamates. Short time later, they applied this concept for the sulfonylation of enol acetates and observed the formation of β-ketosulfones (Scheme 52b) [90]. Again electron-rich, neutral and electron-deficient (hetero)arylsulfonyl chlorides were tolerated under the reported conditions. The scope of enol acetates includes electron-rich and deficient derivatives as well. In addition, also branched enol acetates yield β-ketosulfones.

**Scheme 53:**
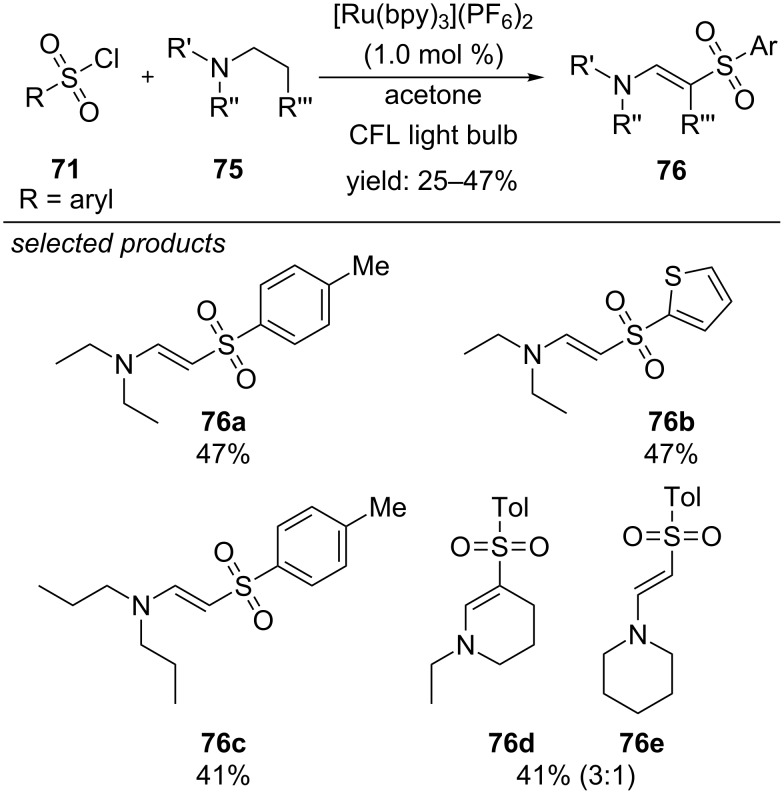
Visible-light photocatalyzed sulfonylation of aliphatic tertiary amines, applying [Ru(bpy)_3_](PF_6_)_2_ as photocatalyst.

A different approach was reported by Zheng and co-workers in 2014: They applied [Ru(bpy)_3_](PF_6_)_2_ as photoredox catalyst and photooxidized tertiary amines under aerobic conditions to the respective radical cationic species (Scheme 53) [91]. Further steps lead to an enamine intermediate, which can react with the former generated sulfonyl radical. They were able to apply a series of electronically and sterically differing (hetero)arylsulfonyl chlorides, affording the desired products in moderate yields. In addition, linear, branched and cyclic tertiary amines were suitable for the reaction.

**Scheme 54:**
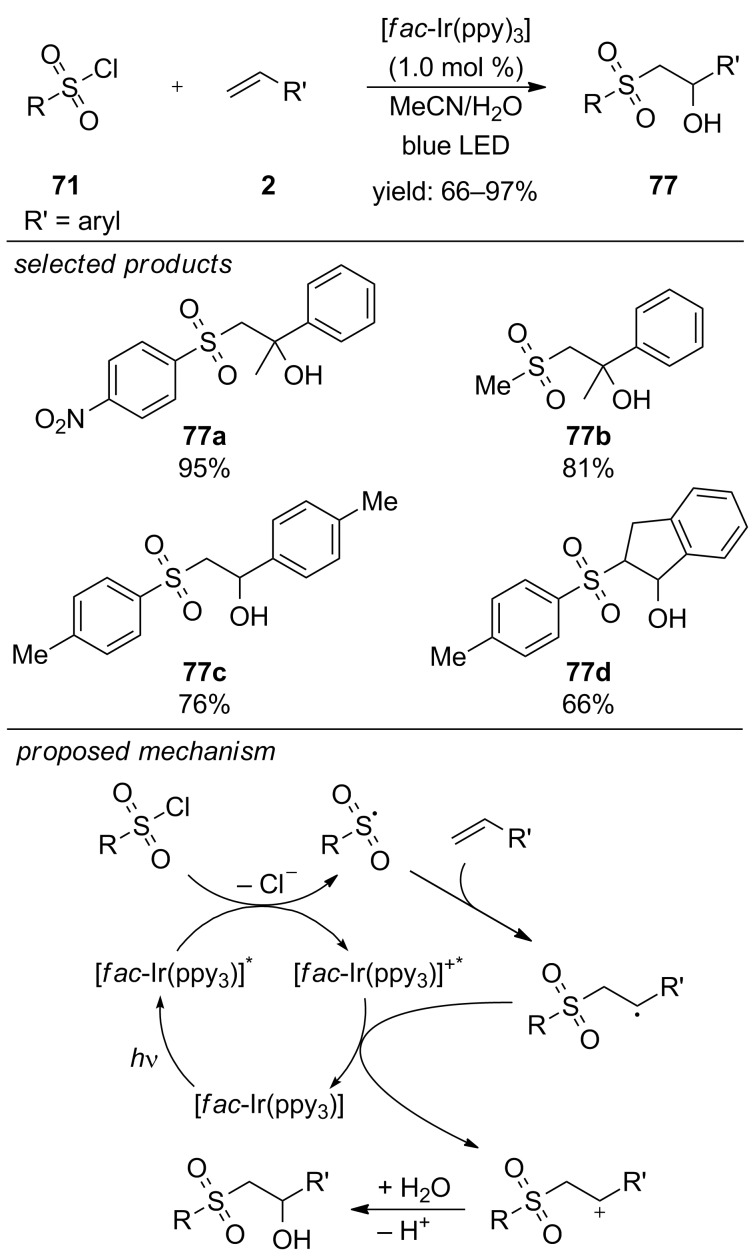
Reiser’s visible-light photoredox-catalyzed preparation of β-hydroxysulfones from sulfonyl chlorides and alkenes.

In 2016, Reiser and co-workers published a new method for the preparation of β-hydroxysulfones from sulfonyl chlorides and alkenes (Scheme 54) [92]. The reaction is catalyzed by [*fac*-Ir(ppy)_3_] under visible-light irradiation and proceeds via an oxidative quenching cycle, generating reactive sulfonyl radicals from sulfonyl chlorides. The key to β-hydroxylation is the use of a mixture of acetonitrile and water (5:1) as solvent. They confirmed by ^18^O-labelling experiments that the oxygen atom in the product originates from the aqueous solvent mixture. This method tolerates electron-rich and electron-deficient arylsulfonyl chlorides. Even sterically demanding 2-mesitylene or 2-naphthylsulfonyl chlorides were suitable for the reaction, affording high yields of the desired products. Furthermore, various styrene derivatives were converted into β-hydroxysulfones.

**Scheme 55:**
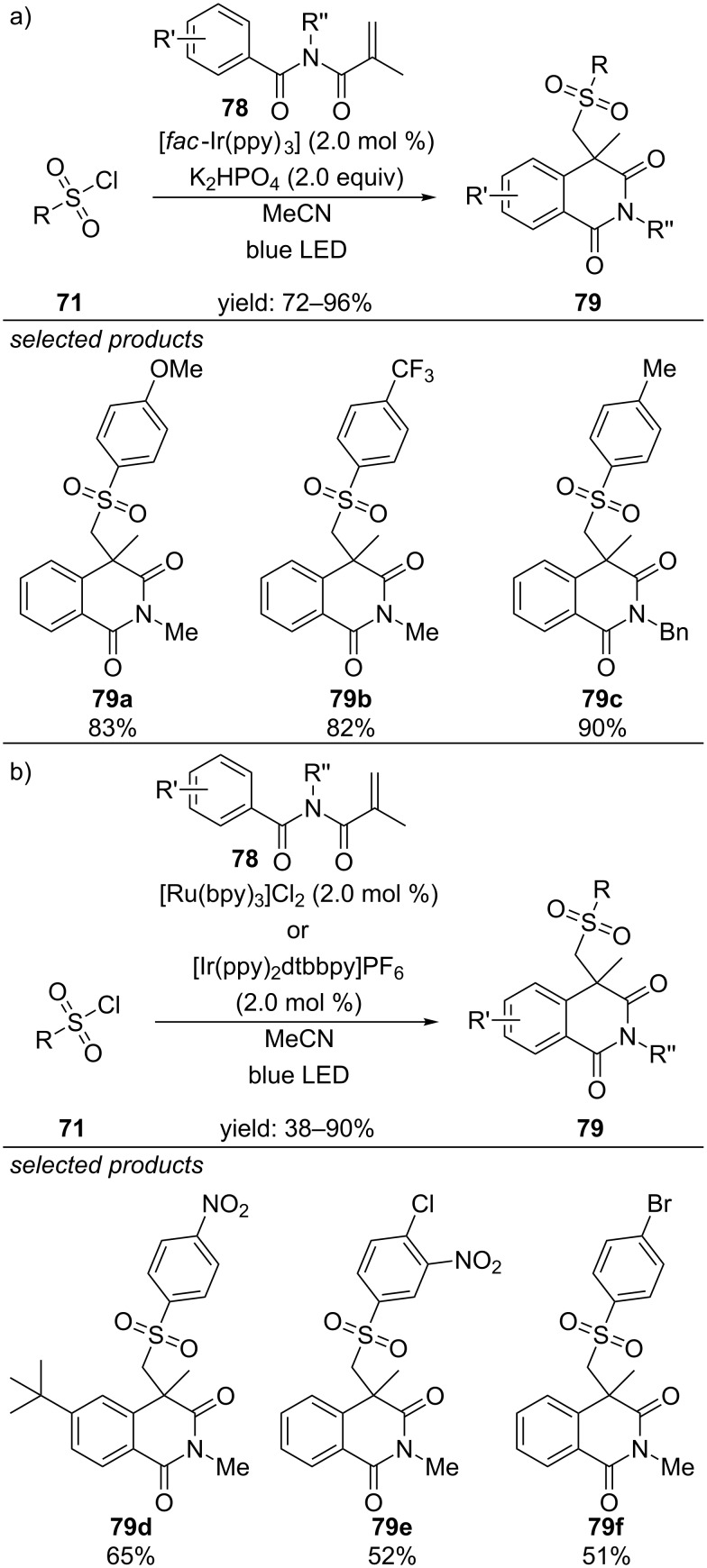
a) Sun’s visible-light-catalyzed approach for the preparation of isoquinolinonediones, applying [*fac*-Ir(ppy)_3_]. b) Xia’s procedure, applying [Ru(bpy)_3_]Cl_2_ or [Ir(ppy)_2_dtbbpy]PF_6_ as photoredox catalysts.

Two different groups simultaneously reported new methods for the preparation of sulfonylated isoquinolinonediones via visible-light photocatalyzed conditions with arylsulfonyl chlorides as sulfonylation agents (Scheme 55) [93–94]. Both reported procedures use *N*-alkyl-*N*-methacryloylbenzamides as precursors. Sun and co-workers utilized [*fac*-Ir(ppy)_3_] as photoredox catalyst, whereas the group of Xia applied either [Ru(bpy)_3_]Cl_2_ or [Ir(ppy)_2_dtbbpy]PF_6_. Both reactions are proposed to proceed via oxidative quenching of the excited state of the photocatalyst. The scope of both procedures is quite similar, including arylsulfonyl chloride derivatives, bearing electron-donating and withdrawing substituents. It is noteworthy, that alkylsulfonyl chlorides react in Sun’s approach. The scope of *N*-alkyl-*N*-methacryloylbenzamides is similar and includes alkyl substituents on the nitrogen atom and electron-rich and poor aromatic moieties.

**Scheme 56:**
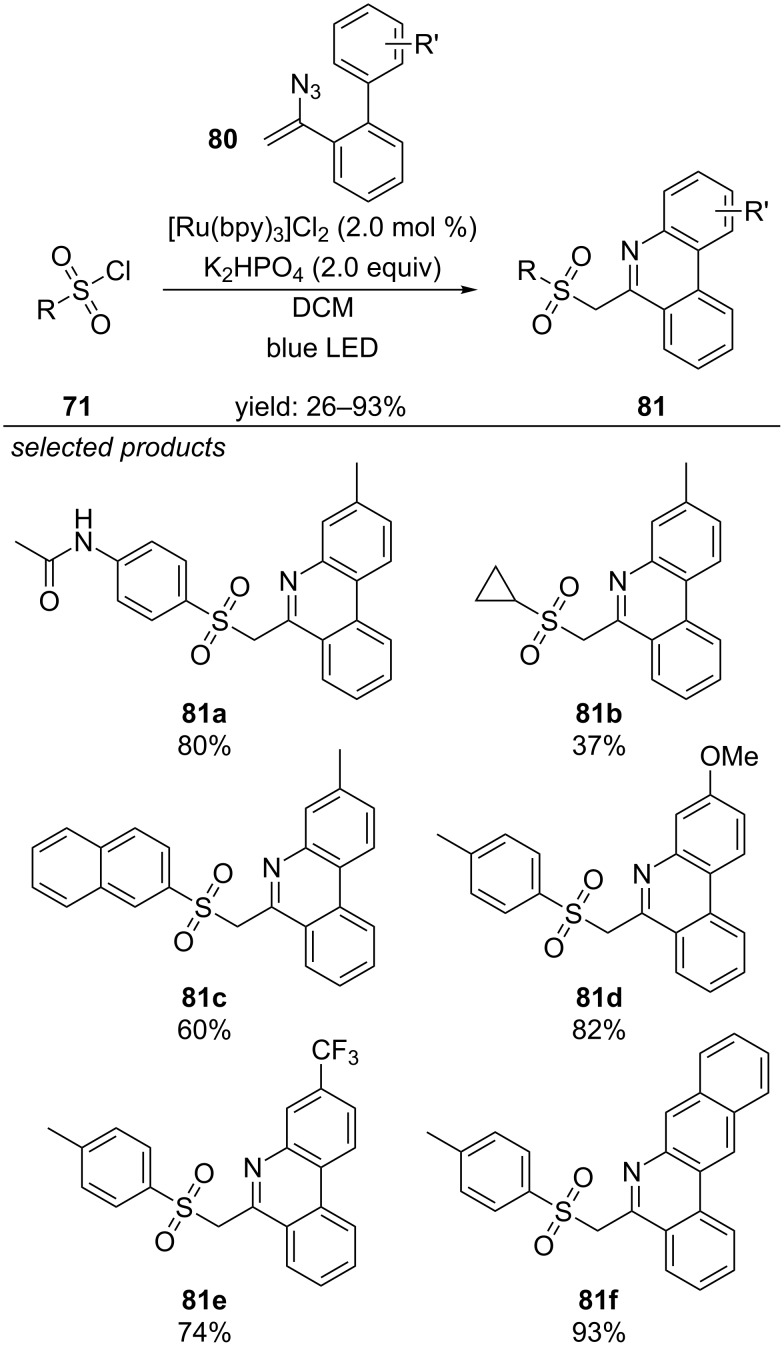
Visible-light photocatalyzed sulfonylation/cyclization of vinyl azides, applying [Ru(bpy)_3_]Cl_2_ as photocatalyst.

Mao and Zhou applied vinyl azides for the radical cross-coupling with sodium sulfinate salts (Scheme 56) [95]. They observed that the azide moiety decomposes during the reaction (loss of N_2_) which triggers an intramolecular cyclization reaction via a nitrogen-centred radical intermediate to form sulfonylated phenanthridines. The reaction is photocatalyzed by [Ru(bpy)_3_]Cl_2_ under irradiation with blue LEDs and proceeds smoothly for a variety of substituted vinyl azides and sulfonyl chlorides.

**Scheme 57:**
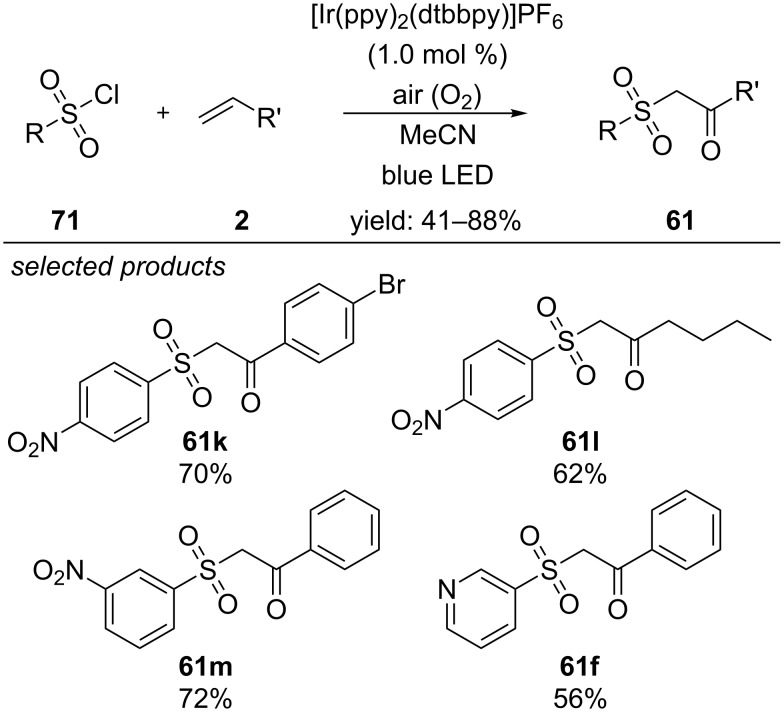
Visible-light photocatalyzed procedure for the formation of β-ketosulfones from aryl sulfonyl chlorides and alkenes.

Recently, the Niu group described visible-light photocatalyzed syntheses of β-ketosulfones, using [Ir(ppy)_2_(dtbbpy)]PF_6_ as catalyst and aerobic oxygen as oxidant under irradiation with blue light (Scheme 57) [96]. They successfully cross-coupled different aliphatic, aromatic and heteroaromatic terminal alkenes with a row of arylsulfonyl chlorides. They propose an oxidative quenching cycle, where the arylsulfonyl chloride is reduced by the excited-state of the photocatalyst, resulting in an arylsulfonyl radical and the oxidized photocatalyst, respectively. After radical-addition to the alkene moiety, aerobic oxygen adds to the carbon-centred radical intermediate, forming a peroxo-radical intermediate. The authors suggest that another equivalent of the radical-addition product couples to the peroxo-radical intermediate and forms a peroxide dimer. Upon homolytic cleavage of the oxygen–oxygen bond and 1,2-hydrogen atom shift, the oxidized photocatalyst is regenerated by oxidizing the resulting C-centred radical intermediate to the respective cation. Deprotonation yields the desired β-ketosulfone.

**Scheme 58:**
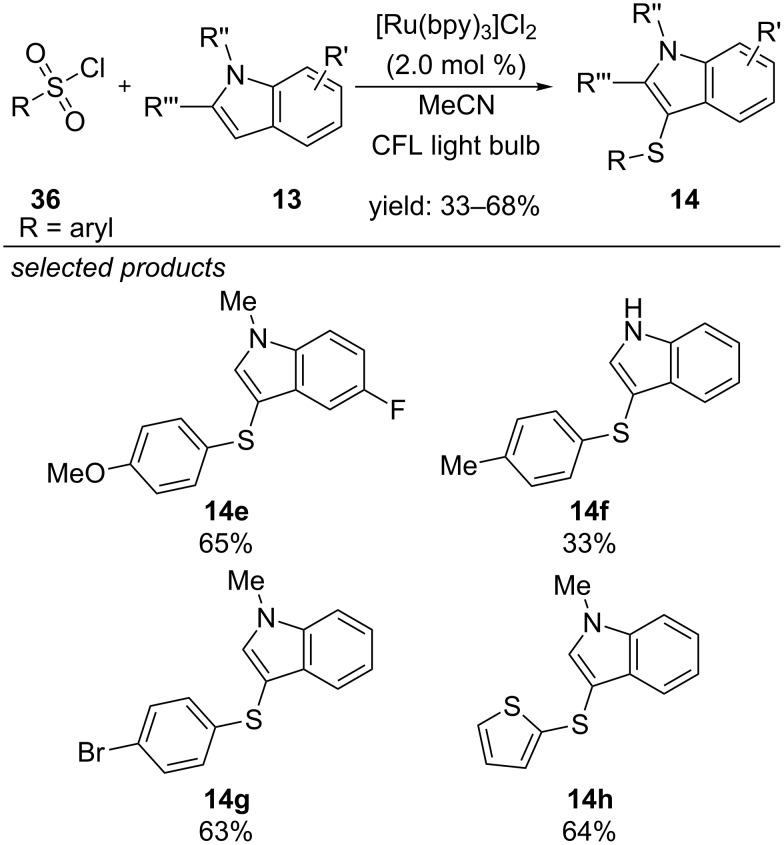
Zheng’s method for the sulfenylation of indole derivatives, applying sulfonyl chlorides via visible-light photoredox catalysis.

A very different approach was reported by Zheng and co-workers in 2012, starting from arylsulfonyl chlorides and using [Ru(bpy)_3_]Cl_2_ as photoredox catalyst. (Scheme 58) [97]. Applying arylsulfonyl chlorides, the sulfonyl moiety was reduced to the respective sulfenyl moiety by an unknown mechanism during the reaction, resulting in sulfenylated *N*-methylindoles after cross-coupling with the respective indole derivatives. The authors propose a reductive quenching cycle, where indole is oxidized by photoexcited [Ru(bpy)_3_]^2+*^, generating the strongly reducing [Ru(bpy)_3_]^+^.

### Sulfonyl hydrazines

#### Formation of sulfone derivatives

**Scheme 59:**
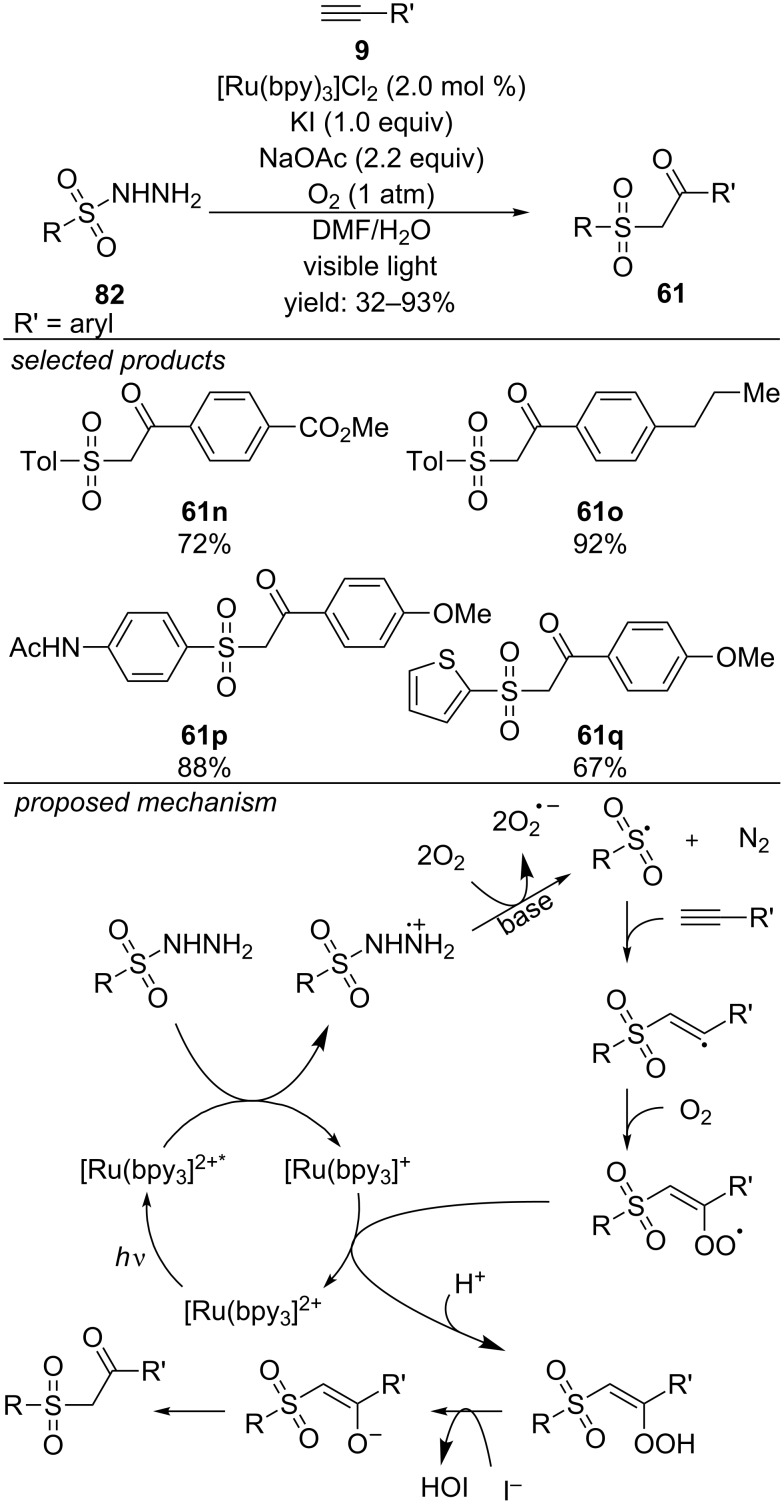
Cai’s visible-light induced synthesis of β-ketosulfones from sulfonyl hydrazines and alkynes.

Recently, Cai and co-workers reported the visible-light catalyzed synthesis of sulfone derivatives, applying sulfonyl hydrazines as sulfur-containing precursors. Similar to the photoreduction of sulfonyl chlorides generating sulfonyl radicals, sulfonyl hydrazines can be photooxidized yielding the respective sulfonyl radical after deprotonation and loss of N_2_. The method was applied for the preparation of β-ketosulfones from alkynes with [Ru(bpy)_3_]Cl_2_ as photoredox catalyst (Scheme 59) [98]. Electron-rich alkynes gave slightly higher yields compared to electron-deficient ones, probably due to a better stabilization of the vinyl radical intermediate, which is generated during the reaction.

**Scheme 60:**
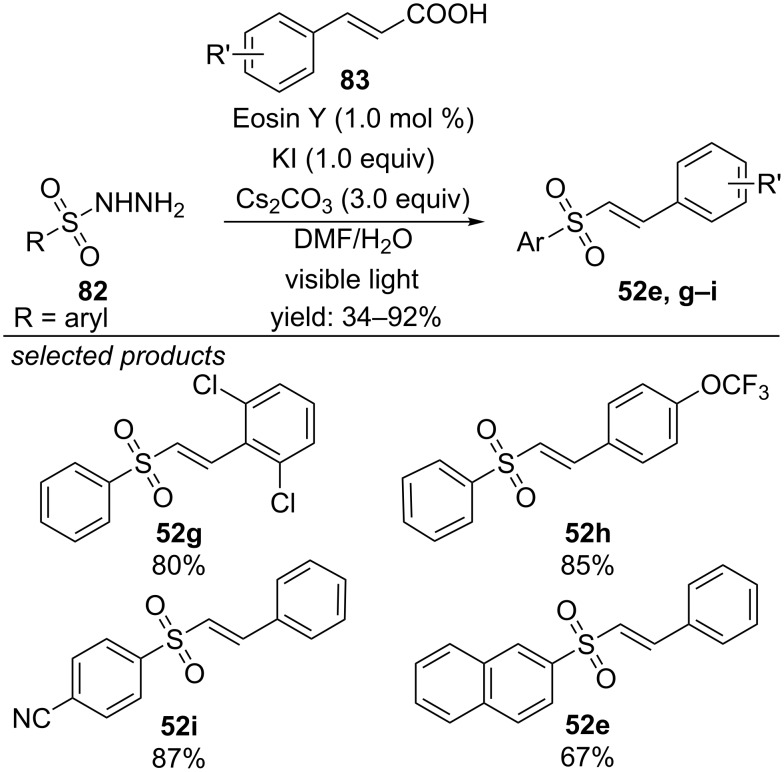
Photoredox-catalyzed approach for the preparation of vinyl sulfones from sulfonyl hydrazines and cinnamic acids.

Photocatalytically generated sulfonyl radicals were also reacted with cinnamic acid derivatives (Scheme 60) [99]. Subsequent decarboxylation leads to vinyl sulfones. Eosin Y is applied as organic photocatalyst and especially electron-deficient cinnamic acid derivatives worked well under the reported conditions.

### Sulfurdioxide

#### Formation of arylsulfonyl chlorides

**Scheme 61:**
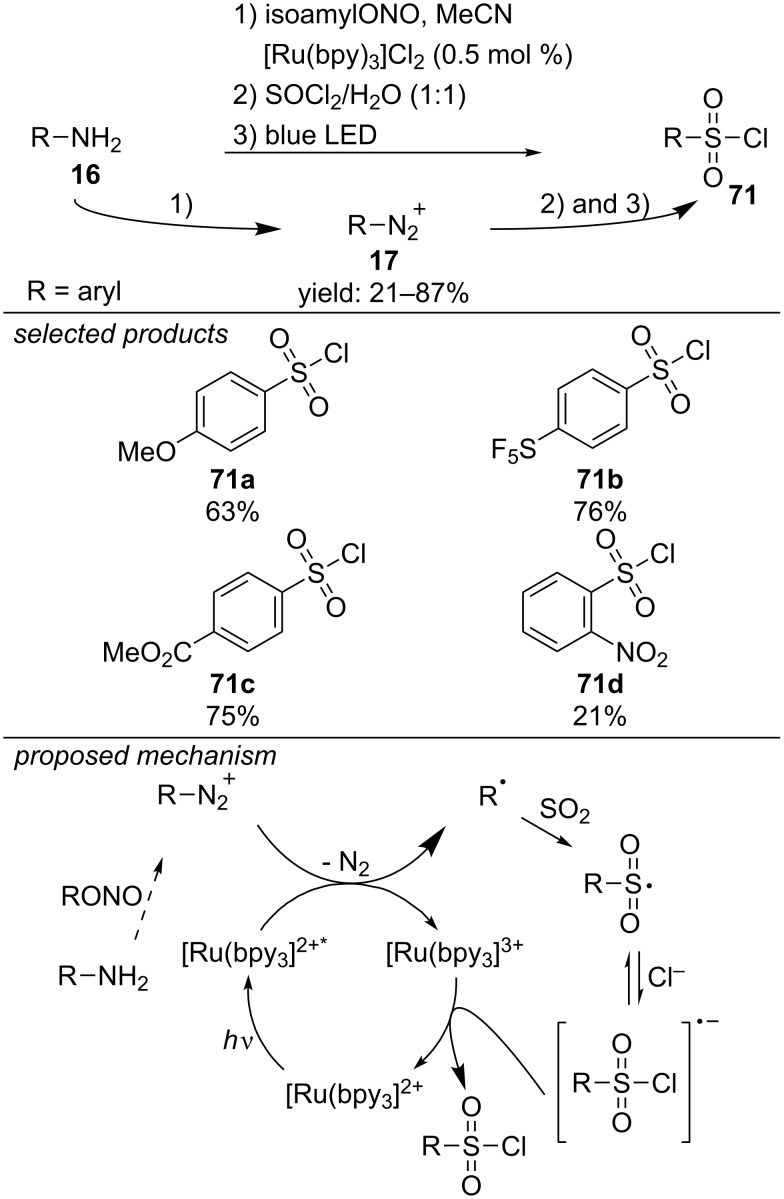
Jacobi von Wangelin’s visible-light photocatalyzed chlorosulfonylation of anilines.

The group of Jacobi von Wangelin developed a synthesis of arylsulfonyl halides from the respective aniline derivatives (Scheme 61) [100]. Aryl diazonium salts are generated in situ from the respective aniline derivatives and photoreduced by [Ru(bpy)_3_]Cl_2_ to the aryl radical. Chlorosulfonylation was accomplished by the addition of sulfur dioxide (SO_2_) and reaction with a chloride anion (Cl^−^). To avoid toxic and gaseous reagents, they used thionyl chloride (SOCl_2_) as source for both SO_2_ and HCl, which are released by hydrolysis in aqueous media. With this approach, they were able to obtain a diverse scope of arylsulfonyl chlorides, from electron-rich as well as electron-deficient anilines. Furthermore, they applied this method for the first visible-light photocatalyzed one-pot synthesis of saccharin from the respective aniline derivative.

#### Formation of *N*-aminosulfonamides

**Scheme 62:**
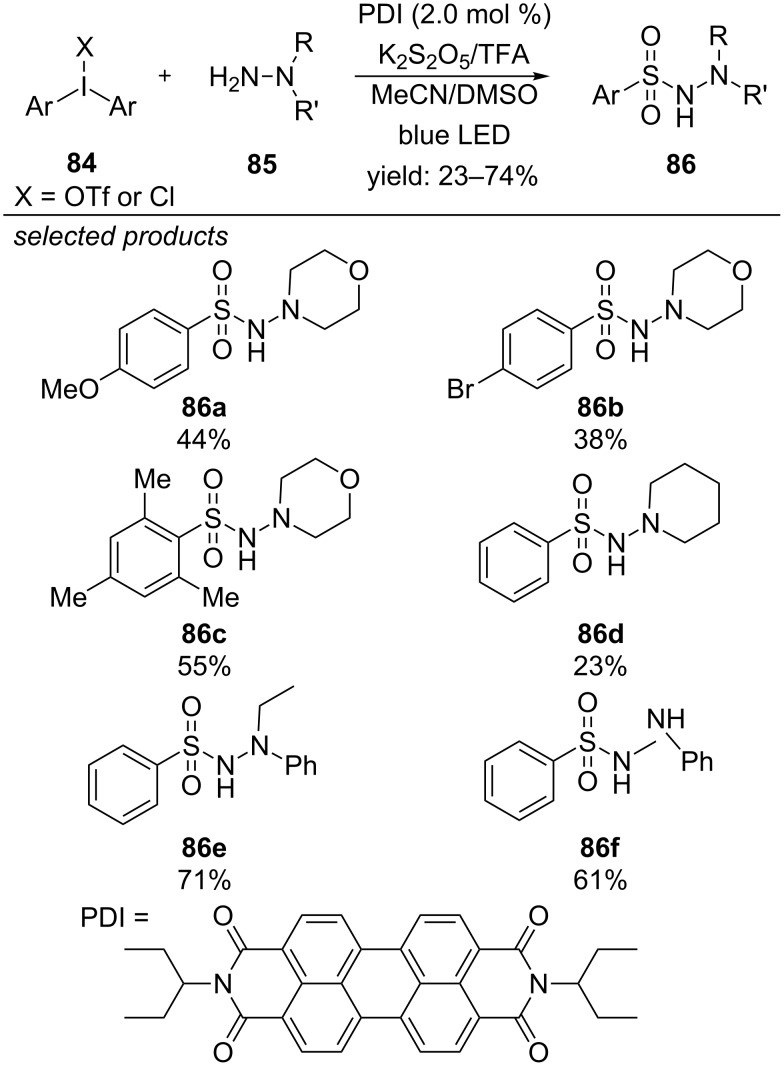
Three-component photoredox-catalyzed synthesis of *N*-amino sulfonamides, applying PDI as organic dye.

The group of Manolikakes successfully substituted gaseous SO_2_ by solid potassium disulfite (K_2_S_2_O_5_), which slowly releases SO_2_ in the presence of trifluoroacetic acid (TFA). They were able to synthesize a series of *N*-aminosulfonamides from the respective diaryliodonium salts, hydrazines and sulfur dioxide in a three-component reaction, applying perylene diimide (PDI) as organic photoredox dye (Scheme 62) [101]. Electron-rich and electron-deficient diaryliodonium salts were tolerated under the presented reaction conditions, affording the desired products in moderate to good yields. Alkyl- and aryl-substituted hydrazine derivatives react with good yields.

#### Formation of sulfones

**Scheme 63:**
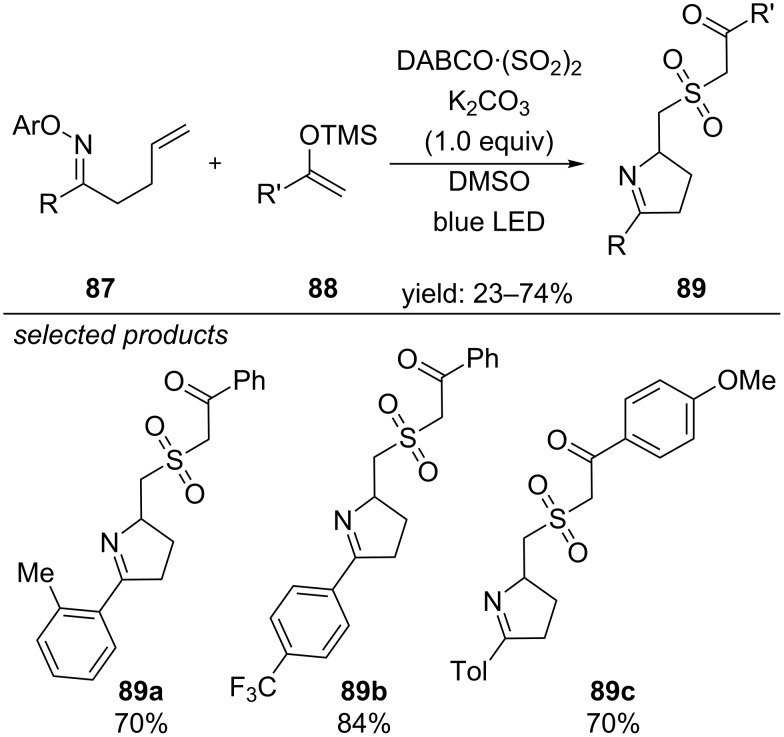
Visible-light induced preparation of complex sulfones from oximes, silyl enol ethers and SO_2_.

Another very recent report from the group of Wu presents a visible-light, photocatalyst-free method for the coupling of oximes and silyl enol ethers via insertion of SO_2_ generating the respective sulfones (Scheme 63) [102]. The authors propose that a donor–acceptor complex is formed by DABCO·(SO_2_)_2_ and the oxime, which absorbs visible light. The reaction is initiated by the formation of a reactive N-radical species, which cyclizes and finally forms the desired product via consecutive addition of SO_2_ and the silyl enol ether.

## Conclusion

Visible-light photoredox catalysis has become a powerful synthetic tool over the last years. Simple and practical reaction set-ups for photocatalysis are easily available, since high power LEDs became a cheap and efficient source of visible-light. As discussed, many photocatalyzed methods were developed for the formation of C–S bonds. Most methods focus on photocatalyzed thiol–ene and thiol–yne reactions for the formation of sulfides, but also methods for the preparation of sulfoxides, sulfones or sulfur-containing heterocycles were reported. The mild conditions of visible light photocatalysis facilitate the late stage functionalization and derivatization of complex and bioactive molecules. In conclusion, photoredox catalysis significantly enriches the synthetic toolbox for the formation of C–S bonds and a wider use in organic synthesis is expected.
